# Structure and polymorphisms of the major histocompatibility complex in the Oriental stork, *Ciconia boyciana*

**DOI:** 10.1038/srep42864

**Published:** 2017-02-17

**Authors:** Hiroki Tsuji, Yukio Taniguchi, Shintaro Ishizuka, Hirokazu Matsuda, Takahisa Yamada, Kazuaki Naito, Hiroaki Iwaisaki

**Affiliations:** 1 Laboratory of Animal Breeding and Genetics, Graduate School of Agriculture, Kyoto University, Kyoto, Japan; 2 Department of Agrobiology, Faculty of Agriculture, Niigata University, Niigata, Japan; 3 Graduate School of Regional Resource Management, University of Hyogo, Toyooka, Japan; 4 Hyogo Park of the Oriental White Stork, Toyooka, Japan; 5 Institute for Research Promotion, Center for Toki and Ecological Restoration, Niigata University, Niigata, Japan

## Abstract

The major histocompatibility complex (MHC) is highly polymorphic and plays a central role in the vertebrate immune system. Despite its functional consistency, the MHC genomic structure differs substantially among organisms. In birds, the MHCs of Galliformes and the Japanese crested ibis (Pelecaniformes) are well-characterized, but information about other avian MHCs remains scarce. The Oriental stork (*Ciconia boyciana*, order Ciconiiformes) is a large endangered migrant. The current Japanese population of this bird originates from a few founders; thus, understanding the genetic diversity among them is critical for effective population management. We report the structure and polymorphisms in *C. boyciana* MHC. One contig (approximately 128 kb) was assembled by screening of lambda phage genomic library and its complete sequence was determined, revealing a gene order of *COL11A2*, two copies of MHC-IIA/IIB pairs, *BRD2, DMA/B1/B2*, MHC-I, *TAP1/2*, and two copies each of pseudo MHC-I and *TNXB*. This structure was highly similar to that of the Japanese crested ibis, but largely different from that of Galliformes, at both the terminal regions. Genotyping of the MHC-II region detected 10 haplotypes among the six founders. These results provide valuable insights for future studies on the evolution of the avian MHCs and for conservation of *C. boyciana*.

The major histocompatibility complex (MHC) is an important region in the vertebrate genome that is involved in defence against infectious diseases and is crucial for adaptive and innate immunity. It is notable for the presence of an extremely high number of polymorphisms of class I and II genes that are responsible for presenting pathogen-derived peptides and for triggering the adaptive immune response^1^.

Polymorphisms in MHC class I and II genes facilitate the binding of diverse pathogens; these evolutionary selection pressures possibly contribute to high genetic variation in MHC loci^2^. Polymorphisms in MHCs are not restricted to allelic variation. Molecular evolution of the MHC involves frequent gene duplication and gene loss resulting in vast rearrangements and pronounced variations in gene number and genomic organization among organisms^3^,^4^.

Since the discovery of the mouse MHC in 1936, MHCs of several mammalian species have been characterized. However, avian MHCs are not well characterized. The genomic organization of the MHCs, spanning the class I and II regions, has been reported in only six members of the order Galliformes, namely the domestic chicken (*Gallus gallus*)^5^, Japanese quail (*Coturnix coturnix japonica*)^6^, domestic turkey (*Meleagris gallopavo*)^7^, golden pheasant (*Chrysolophus pictus*)^8^, black grouse (*Tetrao tetrix*)^9^, and greater prairie-chicken (*Tympanuchus cupido*)^10^, and a Pelecaniformes, the Japanese crested ibis (*Nipponia nippon*)^11^,^12^.

The chicken MHC (also known as MHC-B or B-complex) has a remarkable structure, referred to as the “minimal essential MHC”, which consists of only 19 genes spanning a 92 kb region^5^. The overall structure of MHC-B in five other galliform species is largely similar to that in chicken, whereas gene number, order, and orientation in these structures vary among species^9^. In contrast, the genomic structure of MHC class II in *N. nippon* is considerably different from that in chicken^11^,^12^. For example, the MHC-IIB gene is flanked by the MHC-IIA gene and the MHC IIA/IIB gene pair is located between *COL11A2* and *BRD2* in *N. nippon* whereas, the chicken MHC-B contains no MHC-IIA gene (*BLA*) and two MHC-IIB genes (*BLB1* and *BLB2*) are located on both sides of the *Tapasin* gene^5^. These results suggest that there is large variability in MHC genomic organization among avian species at the order level. Because taxonomic and genomic characterization of avian MHC regions has been limited, it is unclear whether the minimal essential MHC represents the ancestral condition or whether it is a highly derived condition unique to Galliformes. To determine the long-term evolutionary history of the avian MHC, more data of genomic structures from other avian species are required.

The Oriental stork (*Ciconia boyciana*, Order: Ciconiiformes) is a large migrant bird, listed as “endangered” in the 2015 International Union for Conservation of Nature Red List of Threatened Species (http://www.iucnredlist.org). Historically, the Oriental stork inhabited the Far East; habitat loss and overhunting caused a drastic decline in the number of storks, resulting in the extinction of Japanese wild population in 1971. The Oriental stork was designated as a special natural monument in 1956 and its conservation has been carried out for more than 50 years under the Law for the Protection of Cultural Properties in Japan. Captive-breeding programs have been conducted at the Hyogo Park of the Oriental White Stork since 1965. The several Oriental storks surviving in the wild in Japan were captured and used for the breeding; such captive-breeding had, however, been unsuccessful. The first successful captive-breeding was performed in 1989 using six wild birds provided by Russia in 1985. Thereafter, 19 Oriental storks were introduced from Russian nature reserves and some zoos in Japan as founders. The current size of the captive-breeding population in Japan is approximately 95 birds. A project to release *C. boyciana* into the wild was launched in 2005, and in May 2007, one chick hatched in the wild—the first of this species born in the wild in 40 years^13^.

Molecular ecology studies have shown that, in addition to adaptive immune responses, the MHC genotype influences the patterns of mate choice, local adaptation, and expression of sexually selected ornaments^14^,^15^,^16^,^17^. Therefore, understanding the diversity of MHCs is fundamental to the conservation of endangered species. The characterization of the MHC region in *C. boyciana* may provide valuable information about the ancestral avian MHCs. In addition, insights into the genetic diversity of this genomic region could be vital to the conservation of the *C. boyciana* population.

In the present study, we investigated the genomic organization and polymorphisms in the MHC region among the six dominant founders of the Japanese *C. boyciana* population.

## Results

### Genomic structure of the MHC region

**Figure 1:**
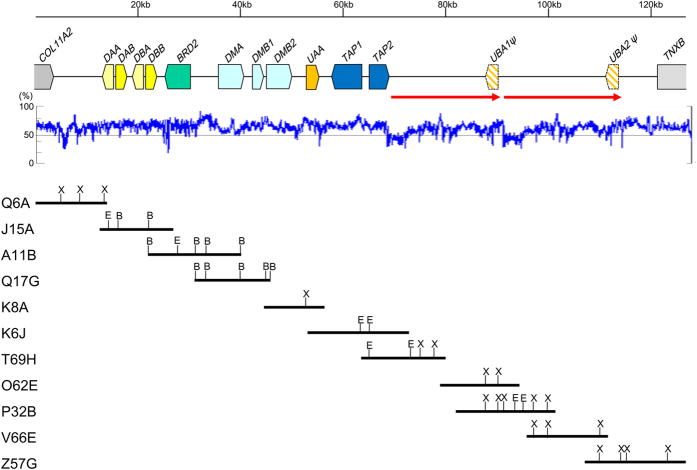
Genomic organization of the Oriental stork MHC. A single contig was assembled. *Collagen-type XI α-2 (COL11A2*), two copies of MHC IIA/IIB pairs (*DAA/DAB* and *DBA/DBB*), *BRD2, DMA, DMB1, DMB2*, MHC I (*UAA*), *TAP1, TAP2*, two copies of pseudo MHC-I (*UBA1ψ* and *UBA2ψ*), and *TNXB* genes and their orientations are indicated. The GC content below the map was calculated by continuous 100-bp windows. Solid bars represent the locations of the isolated lambda phage clones consisting of minimum tailing path. B, E, and X represent restriction sites used for subcloning of *Bam*HI, *EcoR*I, and *Xho*I, respectively. Red arrows below the map represent very long repeat sequences containing the pseudo MHC-I gene.

**Figure 2:**
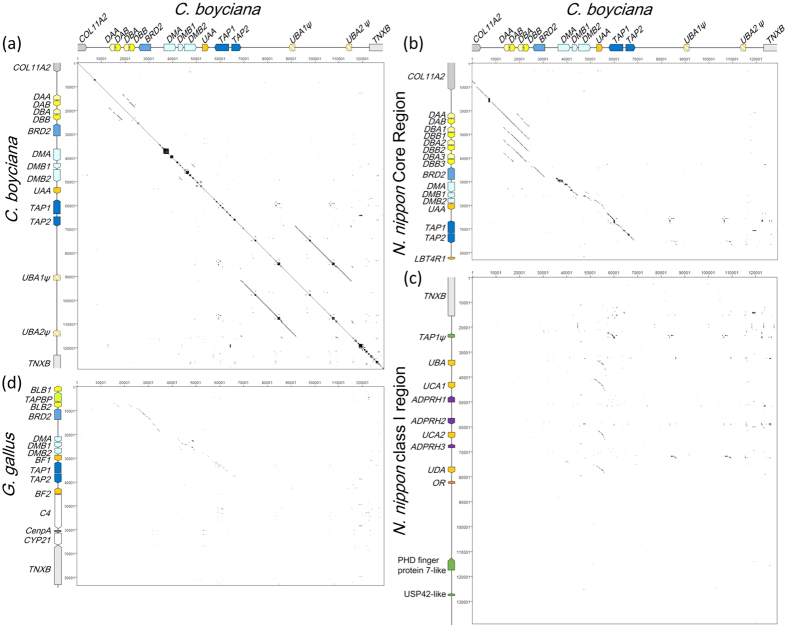
Dot-matrix analysis of the MHC regions. Oriental stork vs. Oriental stork (**a**), Japanese crested ibis core region (**b**), Japanese crested ibis class I region (**c**), and chicken MHC region (**d**). In the chicken MHC, sequences from *BLB1* to *TNXB* were used. The diagonal lines indicate regions where contiguous sequences aligned.

To determine the genomic structure of the Oriental stork MHC region, we screened a lambda phage genomic library. One contig was assembled (Fig. 1; all the isolated phage clones are shown in Fig. S1), and its complete sequence (128,536 bp) was determined. The sequence analysis revealed that this contig contained a partial copy of *COL11A2*, two copies of MHC-IIA/IIB pairs (*DAA/DAB* and *DBA/DBB*), *BRD2, DMA, DMB1, DMB2*, MHC I (*UAA*), *TAP1, TAP2*, two copies of pseudo MHC-I (*UBA1ψ* and *UBA2ψ*), and a partial copy of *TNXB* (Fig. 1). The overall GC content was about 62.4%. The core region (*COL11A2* to *TAP2*) had slightly higher GC content (64.9%) compared to the class I region (59.6%). The analysis with RepeatMasker program identified 120 repetitive elements (47 CR1/LTR repeats, 55 low complexity repeats, and 18 simple repeats) in the 128-kb MHC. The MHC-IIA and IIB genes were arranged in a head-to-head fashion. In two MHC-I loci (*UBA1ψ* and *UBA2ψ*), highly conserved exon 3 and 4 sequences were detected but exon 1 and 2 were absent. The exon 3 sequences in *UBA1ψ* and *UBA2ψ* contained a premature stop codon (Figs S2 and S3). These results suggested that *UBA1ψ* and *UBA2ψ* were pseudogenes. Moreover, the sequence of the DNA fragment containing *UBA1* (approximately 23 kb) was almost identical to that of *UBA2* (Figs 2a and S2), indicating that the two regions were very long tandem repeats. Although the number of tandem repeats containing the pseudo MHC-I gene was estimated to be two by comparing with short sequences (540 bp) from nine phage clones (O19R, O62E, P32B, Z34D, S14A, X65K, V66E, Z57G, and L57C) (Figs S1 and S4), we cannot exclude the possibility that the number of tandem repeats were three or more; further analysis would be necessary to resolve this.

### Comparison of the genomic structure of avian MHC

The dot-matrix analysis revealed that the MHC class II regions (*COL11A2* to *BRD2*) of the Oriental stork had complete synteny to those of the Japanese crested ibis (Fig. 2b), but they were much different from those of chicken (Fig. 2d). The MHC-IIA/IIB gene pair was located between *COL11A2* and *BRD2* in the Oriental stork and the Japanese crested ibis, whereas the chicken MHC-B has been shown to contain no MHC-IIA gene (*BLA*) and two MHC-IIB genes (*BLB1* and *BLB2*) were located on both the sides of *Tapasin* gene^5^. In contrast to the MHC class II region, the central region (*BRD2* to *TAP2*) was highly conserved with perfect syntenic gene order between the three avian species (Fig. 2b and d). The genomic structures of the MHC class I region (*TAP2* to *TNXB*) were different in each species. Whereas the *UAA* locus in the Oriental stork showed high sequence similarities to four MHC-I loci (*UBA, UCA1, UCA2*, and *UDA*) in the Japanese crested ibis^11^, sequence similarity in the class I region containing *UBA1ψ* and *UBA2ψ* was not detected between the two avian species (Fig. 2c). Although some additional genes such as *ADPRH, OR*, and *LBT4R* were detected in the Japanese crested ibis MHC class I region^11^, these genes were not observed in the Oriental stork. The *C4, CenpA*, and *CYP21* genes were located between the MHC class I (*BF2*) and *TNXB* genes in the chicken^18^, but these genes were not detected in the Oriental stork MHC cloned in the present study (Fig. 2d).

### Genetic diversity of the MHC class II region among the six founders

**Figure 3:**
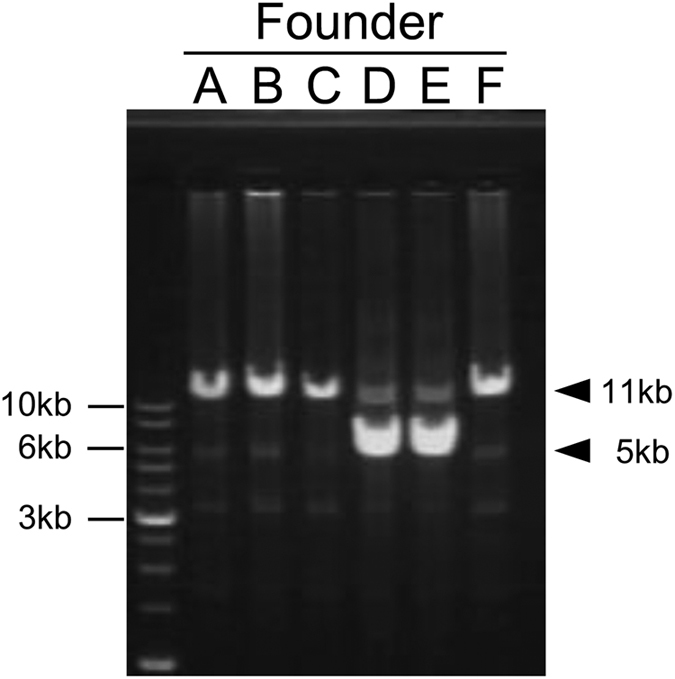
Amplification of the MHC-IIB gene locus from the six founders. Genomic fragments containing both the *DAB1* and *DAB2* loci were amplified from the six founder genomes (A–F) and analysed by 0.7% agarose gel electrophoresis. A single PCR product (approximately 11 kb) was detected in founders A, B, C, and F, but two PCR products (approximately 5 and 11 kb) were amplified in founders D and E.

To investigate the genetic diversity of the MHC class II region among the six dominant founders, the genomic fragments containing both the *DAB* and *DBB* loci were amplified. The PCR products of approximately 11 kb were detected in the founders A, B, C, and F (Fig. 3). In addition to the 11-kb fragment, PCR products of approximately 5 kb were detected in the founders D and E, suggesting that shorter fragments represented alleles with one copy of MHC-IIA/IIB pair.

**Figure 4:**
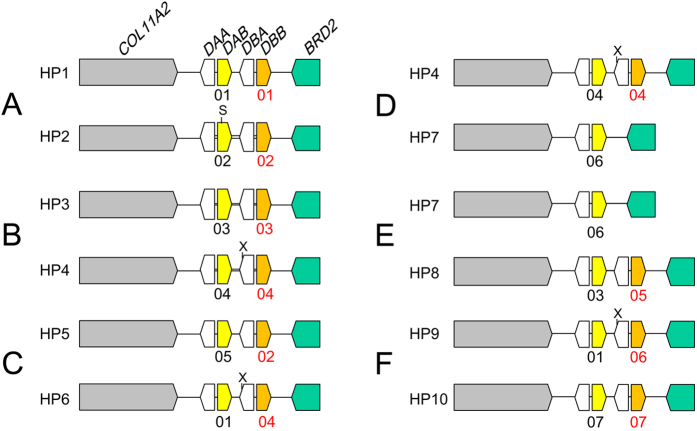
Haplotypes of the MHC class II region in the six founders. PCR products containing both the *DAB1* and *DAB2* loci from the six founders (**A**–**F**) were cloned and two positive clones representing the different alleles were selected from each founder. The exon 2 sequences of both the *DAB1* and *DAB2* genes in the positive clones were determined. Eight and seven exon 2 alleles were detected in *DAB1* and *DAB2* genes, respectively. Black and red numbers below the maps designate the different alleles of *DAB1* and *DAB2* exon 2, respectively. The haplotypes of the MHC class II were decided by the combination of *DAB1* and *DAB2* exon 2 sequences within a single clone. S and X represent restriction sites of *Sal*I and *Xho*I, respectively, used for distinguishing two alleles within a single individual.

The sequencing of PCR products revealed seven exon 2 alleles in the *DAB* locus and seven exon 2 alleles in the *DBB* locus among the six founders (Figs 4 and S5). The haplotypes of the MHC class II region were determined by combination of the *DAB1* and *DAB2* exon 2 alleles within a single PCR product. As a result, 10 haplotypes were detected in the six founders (Fig. 4). The MHC class II genotypes of the founders A, B, C, D, E, and F were estimated to be *hp1/2, hp3/4, hp5/6, hp4/7, hp7/8*, and *hp9/10*, respectively.

### Phylogenetic analysis

Two MHC class II gene loci (*DAA/DAB* and *DBA/DBB*) had high sequence identities in each exon and intron, except in intron 1 (Table 1 and Fig. 2). The intron 1 sequence in the MHC-IIB gene was most divergent in length and identities among the representatives of 18 orders.

**Figure 5:**
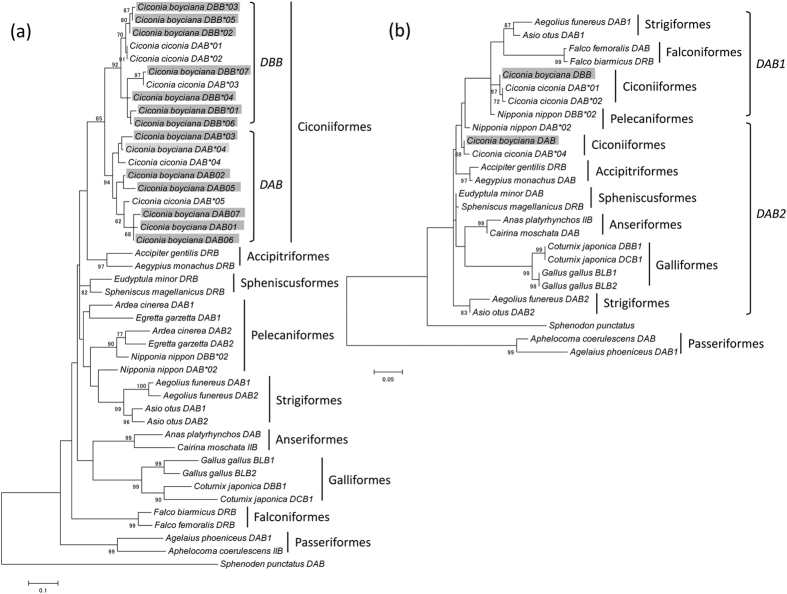
Maximum-likelihood tree with MHC-IIB exon 2 (**a**) or exon 3 (**b**) sequences. In both the analyses, the bootstrap values were evaluated with 1000 replications. Bootstrap values > 60 are shown in this tree. The branch lengths represent the number of changes per site. The Oriental stork sequences are shaded. The accession numbers of the analysed species are as follows: *Accipiter gentilis*, EF370953; *Aegolius funereus*, EF641252, EF641253; *Aegypius monachus*, EF370954; *Agelaius phoeniceus*, AF030997; *Anas platyrhynchos*, AF390589; *Aphelocoma coerulescens*, U23958; *Ardea cinerea*, HM991018, HM991041; *Asio otus*, EF641223, EF641224; *Cairina moschata*, DQ490138; *Ciconia ciconia*, KJ162449–KJ162453; *Coturnix japonica*, AB078884; *Egretta garzetta*, HM991035, HM991056; *Eudyptula minor*, AB302187; *Falco biarmicus*, EF370989; *Falco femoralis*, EF370988; *Gallus gallus*, AL023516; *Nipponia nippon*, AB872443; *Spheniscus magellanicus*, AB325529; *Sphenodon punctatus*, DQ124231.

The phylogenetic relationships of the MHC-IIB genes within two Ciconiformes as well as among nine avian orders were analysed using exon 2 or exon 3 sequences. The maximum-likelihood (ML) tree constructed using MHC-IIB exon 2 sequences showed that the sequences tended to cluster within the orders (Fig. 5a). Within the Ciconiformes cluster containing 14 alleles from the Oriental stork (*C. boycian*a) and five alleles from the White stork (*C. ciconia*)^19^, two subclades representing the *DAB* and *DBB* loci were observed. The alleles from Oriental and White storks were mixed into the same subclade. The ML phylogenetic tree constructed using the partial exon 3 sequences was largely different from that constructed with the exon 2 sequences (Fig. 5b). In the three avian orders Strigiformes, Ciconiiformes, and Pelecaniformes, two MHC-IIB lineages, *DAB1* and *DAB2*, were observed. Whereas sequences from Falconiformes were clustered into the DAB1 clade, sequences from the available single locus in Accipitriformes, Sphenisciformes, or Anseriformes, and two loci in Galliformes were grouped into the DAB2 clade. The MHC-IIB exon 3 sequence of lizard (*Sphenoden punctatus*) was used as an outgroup; however, the cluster of Passeriformes branched off before that of the lizard.

**Figure 6:**
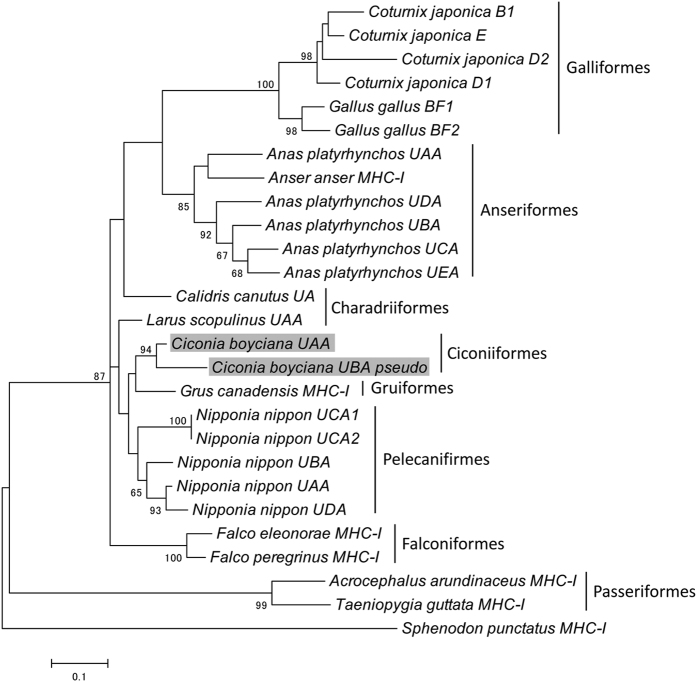
Maximum-likelihood tree constructed using the MHC-I exon 3 sequences. Bootstrap values were evaluated with 1000 replications. Bootstrap values > 60 are shown in this tree. The branch lengths represent the number of changes per site. The Oriental stork sequences are shaded. The accession numbers of the sequences are: *Coturnix japonica*, AB078884; *Gallus gallus*, AL023516; *Anas platyrhynchos*, AY885227; *Calidris canutus*, KC205141; *Larus scopulinus*, HM025953, *Grus canadensis*, AF033106; *Nipponia Nippon*, KP182409; *Falco eleonorae*, JN613263; *Falco peregrinus*, JN613264; *Acrocephalus arundinaceus*, AJ005507; *Taeniopygia guttata*, XM002186531; *Sphenodon punctatus*, DQ145788.

In the ML phylogenetic tree constructed with the MHC-I exon 3 sequences, most of the sequences clustered within the species or orders except those of Charadriiformes (Fig. 6). The two Charadriiformes, *Calidris canutus* and *Larus scopulinus*, were divided into different subclades. The Oriental stork displayed a close relationship with Gruiformes and Pelecaniformes.

## Discussion

The Oriental stork is a critically threatened avian species belonging to the order Ciconiiformes, an avian lineage that is highly divergent from the order Galliformes, but close to the Japanese crested ibis (order Pelecaniformes)^20^. To characterize the Ciconiiformes MHC, we screened a lambda phage genomic library, assembled one contig, and determined its complete sequence. The sequence analysis revealed that the Oriental stork MHC is very compact, containing 13 genes within 128-kb range (Fig. 1).

**Figure 7:**
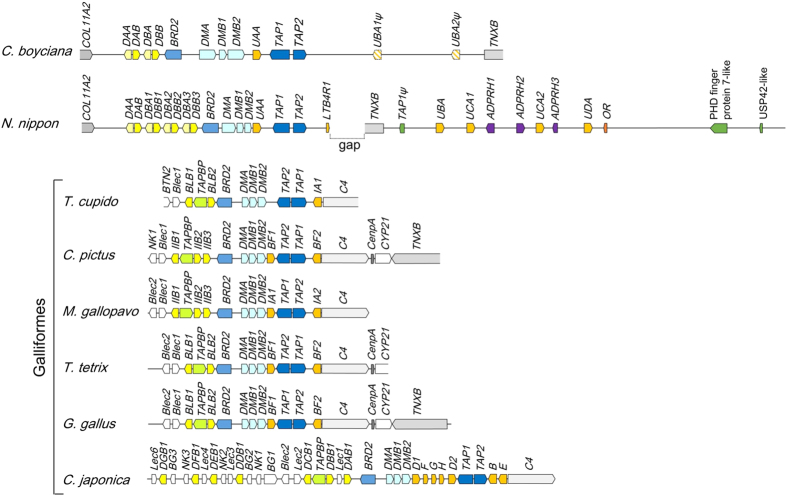
Comparative genomic map of the MHC regions in various avian species. Genomic structures of the MHC regions in *Ciconia boyciana, Nipponia nippon*^11^, and the six Galliformes (*Gallus gallus*^5^, *Coturnix coturnix japonica*^6^, *Meleagris gallopavo*^7^, *Chrysolophus pictus*^8^, *Tetrao tetrix*^9^, and *Tympanuchus cupido*^10^) were compared. In *Nipponia nippon*, the orientation of the fragment containing the MHC-I cluster and *TNXB* remains unknown because of a gap downstream of *TAP2.*

The overall genomic structure of the Oriental stork MHC was close to that of the Japanese crested ibis. The core region (*COL11A2* to *TAP2*) showed complete synteny between the two species (Figs 2b and 7). In contrast, some differences were observed in the class I region; however, the orientation of the fragment containing the MHC-I cluster and *TNXB* remains unknown because of a gap downstream of *TAP2* in the Japanese crested ibis^11^. Although the two MHC-I loci (*UBA1ψ* and *UBA2ψ*) in the Oriental stork were pseudogenes, four MHC-I genes (*UBA, UCA1, UCA2*, and *UDA*) in the Japanese crested ibis were expressed in several tissues, suggesting that these genes were functional. Moreover, the number of the MHC-I genes, insertion of additional genes, and lengths of the intergenic regions were apparently different between the two species.

When comparing Ciconiiformes and Galliformes, the central region (*BRD2* to *TAP2*) was highly conserved, but class II and I regions at both the sides of this region were largely different (Fig. 7). Whereas the structural feature of the MHC class II region in the Oriental stork and the Japanese crested ibis was the gene order of *COL11A2-*MHC-IIA/IIB pairs, *COL11A2* and MHC-IIA genes were not detected in the Galliformes MHC-B. Furthermore, *C4, CenpA*, and *CYP21* were located between MHC class I (*BF2*) and *TNXB* in the Galliformes, but these genes were not detected in the Oriental stork MHC assessed here. Because the gene directions of *TAP2* and *TNXB* were same in the Oriental stork but opposite in the chicken^18^, these might represent the inversion of the genomic fragment containing *C4-CenpA-CYP21-TNXB* between the two avian orders. To resolve this problem, extension of the contig downstream of *TNXB* in the Oriental stork is essential.

Our result suggested that *UAA* locus was the only functional MHC-I gene. The *UAA* locus was proposed to be under coevolution with *TAP1/TAP2* genes due to its close proximity and associated roles in presenting the antigen peptides^21^. Such strong natural selection might be responsible for the highly conserved central MHC region (*BRD2* to *TAP2*). In contrast, the comparison of MHC genomic structures between the three avian orders suggested that the MHC class II and class I regions on either sides of the central region could undergo large diversifications among the avian species under weaker selection pressure.

In the human MHC^22^, the order of genes, which were common between human and avian MHCs, is *COL11A2*, MHC-IIA/IIB pairs (DP), *BRD2, DM*, MHC-I (*HLA-Z*), *TAP*, MHC-IIA/IIB pairs (DQ and DR), *TNXB, CYP21, C4*, and MHC class I region, despite the insertion of many additional genes. Interestingly, although DQ and DR are replaced by the MHC-I cluster in the Oriental stork, the gene order of the Oriental stork MHC is more similar to that of humans compared to that of chicken.

The phylogenetic tree with MHC-IIB exon2 and MHC-I exon3 sequences indicated an avian order- or species-specific clusters (Figs 5 and 6). In contrast, the phylogenetic tree with MHC-IIB exon3 revealed gene-specific clusters (Fig. 5). Burri *et al*.^23^,^24^ suggested two ancestral MHC-IIB lineages (*DAB1* and *DAB2*) from the phylogenetic trees constructed using the owl IIB exon3 sequences. The *DAB* and *DBB* loci in the Oriental stork and the Japanese crested ibis corresponded to *DAB2* and *DAB1*, respectively. These results should support the premise that a unique duplication event, preceding the major avian radiations, produced the two ancestral MHC-IIB lineages. In our phylogenetic tree, made using the first 215 bp of the MHC-IIB exon 3 sequences, the cluster of Passeriformes branched off before that of the lizard, suggesting that the sequence diversities of MHC-IIB gene among the avian orders were much larger. In addition to the phylogenetic analysis of the MHC gene sequences, comparisons between genomic structures of the entire MHC regions could provide novel insights for studies on the evolution of the avian MHC. To elucidate the long-term evolutionary history of avian MHCs, more data on genome structures of whole MHCs from a wide variety of bird species are apparently needed.

Recently, the next-generation sequencing (NGS) technology has been available for *de novo* whole genome sequencing^25^. However, determination of the MHC genomic structure using only the NGS sequencing is difficult owing to factors, such as gene duplication, GC-rich sequences, and several repeat elements. We used a lambda phage genome library in this study; the compact nature of MHC in the Oriental stork enabled us to determine the MHC genomic structure using this library. The protocols for the construction and PCR-screening of lambda phage library are easier than those for BAC or cosmid libraries. Moreover, the use of positive- and negative-selection primers in multiplex PCR for screening is helpful for efficiently isolating a target phage clone. Our strategy for analysing the MHC genomic structure could be effectively applied for the study of many avian species.

As the current Japanese population of the Oriental stork originates from a small numbers of founders, it is very important to know the genetic diversity among the founders for conservation genetics. In this study, 10 haplotypes of MHC class II region were detected among the six dominant founders. Yamamoto *et al*.^26^ reported that nine haplotypes of the mitochondrial D-loop were detected among 26 captive Oriental storks containing founders and their progenies in Japan, and the frequency of haplotypes was comparable to those of the natural populations, such as those of chum salmon and water buffalo. Because the current Oriental stork population in Japan originates not only from the six founders used here but also from a few other founders, the genetic diversity could be considered relatively high. In addition to the polymorphisms of the MHC-IIB exon 2 sequences, the copy number variations (CNV) in the MHC-IIA/IIB pair were observed in the Oriental stork population. The CNVs in the MHC genes have been reported in *N. nippon*^12^ and certain bird species^6^,^27^,^28^,^29^,^30^. These results suggest that CNVs caused by gene duplication, gene loss, and/or gene conversion occur at a relatively high rate within a single bird species.

To the best of our knowledge, this is the first report of MHC genomic organization in Ciconiiformes. This structure was highly conserved with that of the Japanese crested ibis, but was largely different from that of Galliformes. Both the Oriental stork and the Japanese crested ibis are endangered avian species and similar projects for their conservation are being implemented in Japan. However, the recovery of a large population from a small number of founders will be a significant challenge. The current crested ibis population in Japan originates only from five founders; only three MHC class II haplotypes were detected from these^12^. We detected 10 haplotypes of the Oriental stork MHC class II region among the six founders, indicating that the genetic diversity of the MHC region in the Oriental stork population is to some extent larger than that in the Japanese crested ibis population. It would be of further interest to investigate how genetic diversity among founder populations influences the conservation processes. We believe that the structure and polymorphisms of the Oriental stork MHC presented here will provide valuable insights for future studies on the evolution of the avian MHC and for the conservation of the Oriental stork.

## Methods

### Samples

Blood samples from the six Oriental storks (three female founders A, C, and E, and three male founders B, D, and F) were collected for seasonal medical test at the Hyogo Park of the Oriental White Stork (Toyooka, Japan) in 2012. Founders A and B were obtained from Russia in 1985, founders C, D, and E were from a zoo in Japan, and founder F was obtained from Russia in 2003. The sample-collections were performed following a standard protocol by a veterinarian according to the Law for the Protection of Cultural Properties in Japan. The protocols of sample-collections were approved by the Institutional Research Ethics Committee of University of Hyogo. The blood samples leftover after the medical tests were used for this research. The six founders were selected on the basis of their larger contribution to the progeny population. The genomic DNA samples were prepared from the whole blood using the Wizard Genomic DNA Purification kit (Promega) according to the manufacturer’s instructions.

### Primers

The primers and annealing temperatures used for the polymerase chain reaction (PCR) are shown in Table S1. The degenerate primer pairs MHC IIBexon4-F1/R1, TAP1exon7-F1/R1, and TNXB-S1/A1 for amplification of MHC-IIB, TAP1, and TNXB genes, respectively, were designed on the basis of chicken, quail, and crested ibis sequences. Other primers were designed on the basis of sequences determined in this study.

### Construction and screening of genomic library

To determine the genomic structure of the *C. boyciana* MHC region, a genomic library was constructed from founder B. The genomic DNA was partially digested with *Sau*3AI and separated on a 0.5% agarose gel. The digested fragments (15–23 kb) were gel-purified using the Wizard SV Gel and PCR Clean-Up system (Promega), according to the manufacturer’s instructions, and ligated into the Lambda DASH II vector (Stratagene). The ligated DNA mixture was then packaged using Gigapack III Gold packaging extract (Stratagene). The screening was performed using a PCR-based method as previously described^12^. Once a positive phage clone was isolated, sequences from both its ends were determined and used to design primers for obtaining the extended segments in the next round of screening. In some PCR-screenings, positive- and negative-selection primers were used to effectively isolate the target phage clones. All the isolated lambda phage clones and primer pairs used for screening of each phage clone are shown in Figure S1 and Table S2.

Three lambda phage clones (C3D, J15A, and A11B) were isolated by PCR-screening with primer pair MHC IIBexon4-F1/R1 (Figs 1 and S1, Table S2). A lambda phage clone Q6A was isolated by genome walking to the upstream of C3D. A lambda phage clone Q17G was isolated by genome walking to the downstream of A11B.

A lambda phage clone Q13C was isolated by PCR-screening with the primer pair TAP1exon7-F1/R1. Two lambda phage clones (K8A and K6J) were isolated by genome walking to the upstream and downstream of Q13C, respectively. T69H, and subsequently V66E, were obtained by two rounds of genome walking to the downstream of K6J. Five phage clones (O19R, O62E, P32B, S14A, and X65K) were obtained as V66E F/R primer pair positive clones.

A lambda phage clone, Z34D, was isolated with the primer pair MHC-I F/R that was designed on the basis of the MHC-I gene sequence within the clone Q13C. A lambda phage clone, N49F, was isolated by genome walking to the downstream of Z34D.

Two lambda phage clones (L57C and G57G) were isolated by PCR-screening with the primer pair TNXB-S1/A1.

### Analysis of isolated lambda phage clones

The phage DNA from the positive clones was purified, digested with *Bam*HI, *Eco*RI, and/or *Xho*I, and then subcloned into pBluescript II vector (Stratagene) (Figs 1 and S1). The sequences from both the ends of some subclones were determined by using M13 forward and reverse primers and analysed through homology searches using BLAST (http://blast.ncbi.nlm.nih.gov/blast). The sequencing was carried out by Greiner Japan Co., Ltd. (Tokyo, Japan). By combining these results, restriction maps of each lambda phage clone were constructed.

### Complete sequencing

Before completing the construction of contig, the insert DNAs of the subclones from phage clones Q6A, A11B, Q17G, K8A, K6J T69H, V66E, and Z34D (Fig. S1) were purified, mixed, and sequenced with a next-generation sequencer (NGS) HiSeq2500 (Illumina) for 101 cycle in pair-end mode. The sequence data of approximately 1.2 Gb (number of reads: 11,944,902, mean quality score: 33.78) were assembled using the “Velvet” program. The NGS sequencing and assembly were performed at the Hokkaido System Science Co. Ltd. (Sapporo, Japan). However, because the assembled sequence contained several gaps and many ambiguous nucleotides, especially in the repeat sequence regions, the phage clones consisting of minimal tailing paths were selected (Fig. 1) for complete sequencing with a Sanger sequencer. The insert DNAs of subclones from the selected phage clones were further digested with some restriction enzymes and cloned into a pBluescript II vector (Stratagene). The restriction enzymes were chosen on the basis of sequences assembled by NGS. The clones with shorter inserts (<1.2 kb) were sequenced using M13 forward and reverse primers. For clones with longer inserts (>1.2 kb) or regions that were difficult to sequence, nested deletion constructs were prepared using a Deletion Kit for Kilo-Sequencing (TAKARA) and then sequenced. Few of the remaining gaps were sequenced by using specific primers. The sequences were manually assembled using GENETYX version 11 (Software Development). The complete sequence of the Oriental stork MHC region was deposited in the DNA Data Bank of Japan (DDBJ) (accession number: LC180358).

### Gene prediction and sequence analysis

The coding regions within the determined complete sequence were searched using GENSCAN program (http://genes.mit.edu/GENSCAN.html) with the parameters for vertebrates and through homology with the sequences (KP182408 and KP182409) from Japanese crested ibis using BLAST (http://blast.ncbi.nlm.nih.gov/blast). The sequence alignments and dot-matrix analysis were performed using GENETYX using the default parameters. The locus names of MHC class I and II genes follow the nomenclature suggested by Klein *et al*.^31^ and incorporate the information of sequence similarity and gene order in the Japanese crested ibis. Repeat elements were identified using RepeatMasker (www.repeatmasker.org).

### Genetic diversity of MHC class II region among the six founders

To investigate the genetic diversity of the MHC class II region, genomic fragments containing both *DAB1* and *DAB2* loci were amplified from the six founders (A–F) with the primers DAB1int1-F1 and BRD2-R2 (Table S1) that were located in *DAB1* intron 1 and in *BRD2* exon 8, respectively. The polymerase chain reactions were performed by a two-step method (initial denaturation at 95 °C for 5 min, and 35 cycles of denaturation at 95 °C for 30 sec, annealing and extension at 72 °C for 7 min) using KOD FX Neo DNA polymerase (TOYOBO). The PCR products were cloned into pBluescriptII (Stratagene) and two positive clones representing different alleles were selected from each founder. The exon 2 sequences of both *DAB1* and *DAB2* in the positive clones were determined using the primers DAB1int1-F1 and DAB2int1-F1 (Table S1), respectively. The haplotypes of the MHC class II were decided by the combination of *DAB1* and *DAB2* exon 2 sequences within a single clone. The *DAB1* and *DAB2* exon 2 sequences of the Oriental stork were deposited in the DDBJ (accession numbers: LC183878-LC183891).

### Construction of phylogenetic tree

The phylogenetic relationships of the MHC genes among nine avian orders were analysed using MHC-IIB exon 2, exon 3 (first 215 bp), or MHC-I exon 3 sequences. For Ciconiformes, sequences of all the available alleles from the Oriental and White storks^19^ were used. The sequences of lizard (*S. punctatus*) were used as outgroups. The best-fitting nucleotide substitution model for each codon position was evaluated using Find Best DNA/Protein Models (ML) in MEGA version 5.2^32^ according to the Akaike information criterion. A maximum-likelihood tree with MHC-IIB exon 2 or exon 3 sequences was constructed using a Tamura 3-parameter model with gamma distribution in MEGA. A maximum-likelihood tree with MHC-I exon 3 sequences was constructed by using a Kimura 2-parameter model with gamma distribution in MEGA.

## Additional Information

**How to cite this article:** Tsuji, H. *et al*. Structure and polymorphisms of the major histocompatibility complex in the Oriental stork, *Ciconia boyciana. Sci. Rep.* **7**, 42864; doi: 10.1038/srep42864 (2017).

**Publisher's note:** Springer Nature remains neutral with regard to jurisdictional claims in published maps and institutional affiliations.

## Supplementary Material

Supporting Information

## Figures and Tables

**Table 1:** Comparison of exon and intron sequences between *DAA/DAB* and *DBA/DBB* loci.

	MHC-IIA										
	Exon 1	Intron 1	Exon 2	Intron 2	Exon 3	Intron 3	Exon 4				
*DAA*	73 bp	761 bp	258 bp	275 bp	282 bp	106 bp	155 bp				
*DBA*	73 bp	728 bp	258 bp	289 bp	282 bp	106 bp	155 bp				
Identity (%)	100%	65%	81%	71%	94%	100%	97%				
	**MHC-IIB**										
	**Exon 1**	**Intron 1**	**Exon 2**	**Intron 2**	**Exon 3**	**Intron 3**	**Exon 4**	**Intron 4**	**Exon 5**	**Intron 5**	**Exon 6**
*DAB*	91 bp	626 bp	270 bp	254 bp	282 bp	114 bp	107 bp	131 bp	24 bp	168 bp	9 bp
*DBB*	91 bp	272 bp	270 bp	254 bp	282 bp	111 bp	107 bp	131 bp	24 bp	168 bp	9 bp
Identity (%)	47%	86%	100%	95%	96%	100%	100%	100%	100%	100%	98%

## References

[b1] Klein J. Antigen-major histocompatibility complex-T cell receptors: inquiries into the immunological ménage à trois. Immunol. Res. 5, 173–190 (1986). 2437228 10.1007/BF02919199

[b2] Zinkernagel R. M. & Doherty P. C. MHC-restricted cytotoxic T cells: studies on the biological role of polymorphic major transplantation antigens determining T-cell restriction-specificity, function and responsiveness. Adv. Immunol. 27, 51–177 (1979). 92183 10.1016/s0065-2776(08)60262-x

[b3] Kulski J. K., Shiina T., Anzai T., Kohara S. & Inoko H. Comparative genomic analysis of the MHC: the evolution of class I duplication blocks, diversity and complexity from shark to man. Immunol. Rev. 190, 95–122 (2002). 12493009 10.1034/j.1600-065x.2002.19008.x

[b4] Kelley J., Walter L. & Trowsdale J. Comparative genomics of major histocompatibility complexes. Immunogenetics 56, 683–695 (2005). 15605248 10.1007/s00251-004-0717-7

[b5] Kaufman J. . The chicken B locus is a minimal essential major histocompatibility complex. Nature 401, 923–925 (1999). 10553909 10.1038/44856

[b6] Hosomichi K. . The major histocompatibility complex (MHC) class IIB region has greater genomic structural flexibility and diversity in the quail than the chicken. BMC Genomics 7, 322 (2006). 17184537 10.1186/1471-2164-7-322 PMC1769493

[b7] Chaves L. D., Krueth S. B. & Reed K. M. Defining the turkey MHC: sequence and genes of the B locus. J. Immunol. 183, 6530–6537 (2009). 19864609 10.4049/jimmunol.0901310

[b8] Ye Q., He K., Wu S. Y. & Wan Q. H. Isolation of a 97-kb minimal essential MHC B locus from a new reverse-4D BAC library of the golden pheasant. PLoS One 7, e32154 (2012). 22403630 10.1371/journal.pone.0032154 PMC3293878

[b9] Wang B., Ekblom R., Strand T. M., Portela-Bens S. & Höglund J. Sequencing of the core MHC region of black grouse (*Tetrao tetrix*) and comparative genomics of the galliform MHC. BMC Genomics 13, 553 (2012). 23066932 10.1186/1471-2164-13-553 PMC3500228

[b10] Eimes J. A. . Greater prairie chickens have a compact MHC-B with a single class IA locus. Immunogenetics 65, 133–144 (2013). 23179555 10.1007/s00251-012-0664-7

[b11] Chen L. C. . Genomic organization of the crested ibis MHC provides new insight into ancestral avian MHC structure. Sci. Rep. 5, 7963 (2015). 25608659 10.1038/srep07963 PMC4302302

[b12] Taniguchi Y. . Structure and polymorphism of the major histocompatibility complex class II region in the Japanese Crested Ibis, *Nipponia nippon*. PLoS One 9, e108506 (2014). 25247679 10.1371/journal.pone.0108506 PMC4172706

[b13] Naito K. & Ikeda H. Habitat restoration for the reintroduction of Oriental White Storks. Global Environ. Res. 11, 217–221 (2007).

[b14] Von Schantz T., Wittzell H., Goransson G. & Grahn M. Mate choice, male condition-dependent ornamentation and MHC in the pheasant. Hereditas 127, 133–140 (1997).

[b15] Ekblom R. . Spatial pattern of MHC class II variation in the great snipe (*Gallinago media*). Mol. Ecol. 16, 1439–1451 (2007). 17391268 10.1111/j.1365-294X.2007.03281.x

[b16] Hale M. L., Verduijn M. H., Moller A. P., Wolff K. & Petrie M. Is the peacock’s train an honest signal of genetic quality at the major histocompatibility complex? J. Evol. Biol. 22, 1284–1294 (2009). 19453370 10.1111/j.1420-9101.2009.01746.x

[b17] Roberts S. C. Complexity and context of MHC-correlated mating preferences in wild populations. Mol. Ecol. 18, 3121–3123 (2009). 19682244 10.1111/j.1365-294X.2009.04244.x

[b18] Shiina T. . Extended gene map reveals tripartite motif, C-type lectin, and Ig superfamily type genes within a subregion of the chicken MHC-B affecting infectious disease. J. Immunol. 178, 7162–7172 (2007). 17513765 10.4049/jimmunol.178.11.7162

[b19] Burri R., Promerová M., Goebel J. & Fumagalli L. PCR-based isolation of multigene families: lessons from the avian MHC class IIB. Mol. Ecol. Resour. 14, 778–788 (2014). 24479469 10.1111/1755-0998.12234

[b20] Hackett S. J. . A phylogenomic study of birds reveals their evolutionary history. Science 320, 1763–8 (2008). 18583609 10.1126/science.1157704

[b21] Walker B. A. . The dominantly expressed class I molecule of the chicken MHC is explained by coevolution with the polymorphic peptide transporter (TAP) genes. Proc. Natl. Acad. Sci. USA 108, 8396–8401 (2011). 21536896 10.1073/pnas.1019496108 PMC3100931

[b22] Horton R. . Gene map of the extended human MHC. Nat. Rev. Genet. 5, 889–899 (2004). 15573121 10.1038/nrg1489

[b23] Burri R., Hirzel H. N., Salamin N., Roulin A. & Fumagalli L. Evolutionary patterns of MHC class II B in owls and their implications for the understanding of avian MHC evolution. Mol. Biol. Evol. 25, 1180–1191 (2008). 18359775 10.1093/molbev/msn065

[b24] Burri R., Salamin N., Studer R. A., Roulin A. & Fumagalli L. Adaptive divergence of ancient gene duplicates in the avian MHC class II beta. Mol. Biol. Evol. 27, 2360–2374 (2010). 20463048 10.1093/molbev/msq120

[b25] Li R. . The sequence and de novo assembly of the giant panda genome. Nature 463, 311–317 (2010). 20010809 10.1038/nature08696 PMC3951497

[b26] Yamamoto Y. . Determination of the complete nucleotide sequence and haplotypes in the D-loop region of the mitochondrial genome in the oriental white stork, *Ciconia boyciana*. Genes Genet. Syst. 75, 25–32 (2000). 10846618 10.1266/ggs.75.25

[b27] Eimes J. A. . Rapid loss of MHC class II variation in a bottlenecked population is explained by drift and loss of copy number variation. J. Evol. Biol. 24, 1847–1856 (2011). 21605219 10.1111/j.1420-9101.2011.02311.x

[b28] Strandh M. . Major histocompatibility complex class II compatibility, but not class I, predicts mate choice in a bird with highly developed olfaction. Proc. Biol. Sci. 279, 4457–4463 (2012). 22951737 10.1098/rspb.2012.1562 PMC3479804

[b29] Alcaide M. . Extraordinary MHC class II B diversity in a non-passerine, wild bird: the Eurasian Coot *Fulica atra* (Aves: Rallidae). Ecol. Evol. 4, 688–698 (2014). 24683452 10.1002/ece3.974 PMC3967895

[b30] Meyer-Lucht Y. . Adaptive and neutral genetic differentiation among Scottish and endangered Irish red grouse (*Lagopus lagopus scotica*). Conserv. Genet. 17, 615–630 (2016).

[b31] Klein J. . Nomenclature for the major histocompatibility complexes of different species: a proposal. Immunogenetics 31, 217–219 (1990). 2329006 10.1007/BF00204890

[b32] Tamura K. . MEGA5: molecular evolutionary genetics analysis using maximum likelihood, evolutionary distance, and maximum parsimony methods. Mol. Biol. Evol. 28, 2731–2739 (2011). 21546353 10.1093/molbev/msr121 PMC3203626

