# Supplementary material for: Structure and polymorphisms of the major histocompatibility complex in the Oriental stork, Ciconia boyciana
Source: Sci Rep. 2017 Feb 17;7:42864. doi: 10.1038/srep42864 (PMC5314415; doi:10.1038/srep42864)
Supplement: Supporting Information [file srep42864-s1.pdf]

Structure and Polymorphism of the Major Histocompatibility Complex in the Oriental stork, *Ciconia boyciana*

Hiroki Tsuji, Yukio Taniguchi, Shintaro Ishizuka, Hirokazu Matsuda, Takahisa Yamada, Kazuaki Naito, Hiroaki Iwaisaki

Supporting information

Table S1. List of primers used for polymerase chain reaction

Table S2. Primer pairs used for screening of each positive clone

Figure S1. Genomic organization of the Oriental stork MHC and all isolated phagenclones

Figure S2. Comparison of nucleotide sequences of long repeats in MHC class I region

Figure S3. Alignment of amino acid sequences of MHC-I genes (*UAA*, *UBA1 ɸ* and *UBA2 ɸ*)

Figure S4. Alignment of nucleotide sequences of 540 bp from the *Xho*I site among 9 phage clones

Figure S5. Alignment of predicted amino acid sequences of MHC-IIB exon 2 alleles from the 6 founders of Oriental storks in Japan.

Table S1. List of primers used for PCR

Primer name	Sequence 5'-3'	Annealing temperature (°C)	Product size (bp)	Usage	sequence used for primer design
MHC IIBexon4-F1	AGACGGAGCGCGTGGTGTCCAC	63	356		MHC-IIB gene sequence from chicken, quail and crested ibis
MHC IIBexon4-R1	GAAGAGCCCCAGCGCCAGGAAG				
TAP1exon7-F1	GCCCAGGGTAGGAGAACCA	60	176	cloning	TAP1 gene sequence from chicken and quail
TAP1exon7-R1	CTGTCCCCAGGTCCTGCTC				
TNXB-S1	GTGCACCTCCACCCGGTACA	63	288	cloning	TNXB gene sequence from crested ibis
TNXB-A1	GTGGCCCGTGCCATCCTCTC				
C3D-S	AAGTGCTCTGATTCACACCAG	53	290	genome walking	5' terminal sequence of clone C3D
C3D-A	CCAGACAAGGAGCAAATCCC				
A11B-F	CACCATCCACCCAACTCCT	58	386	genome walking	3' terminal sequence of clone A11B
A11B-R	ACCAGTGACGAGTGGTGTC				
A11B BRD2-F	GCCGGAGGTTTCCAACCCCAAG	63	300	genome walking	BRD2 gene sequence within clone A11B
A11B BRD2-R	ACATCCGGGGTCAGGCAGCA				
Q13C-S	ATGTGGGACCCCAGGGGTAGT	62	293	genome walking	5' terminal sequence of clone Q13C
Q13C-A	TGAGCCCCTCAGACCGTCCTT				
MHC I-F	GGGAGGGACTTCATCGCCTT	60	304	cloning, genome walking	MHC-I gene sequence within clone Q13C
MHC I-R	GCCTCCTTCCCCGACACT				
Q13C-F	TCCCCTCCAAGTGCCAGTGA	60	217	genome walking	3' terminal sequence of clone Q13C
Q13C-R	GGCACAAAGTGGGGAGGACAA				
K6J-F	CGTCTACCAAAGGAAACTGCC	57	195	genome walking	3' terminal sequence of clone K6J
K6J-R	TTTTCTTACCGGTTGTGCTCC				
T69H-F	TACCAGCTGGAGCTCTGCC	60	281	genome walking	3' terminal sequence of clone T69H
T69H-R	AGGACAAGGCACCCCAGAAG				
V66E-F	TGTGACTAGTCCTTGCAGCGT	58	201	cloning, genome walking	3' terminal sequence of clone V66E
V66E-R	AGGGCCTTATACTTCCATGGGT				
Z34D-F	GGCAACTCTGCAGACAGCAAG	59	374	genome walking	3' terminal sequence of clone Z34D
Z34D-R	TGGGCTCACTTTCACACCCAA				
DAB1int1-F1	GATGAATTCCTCAGGATCGCACCGCCAGTGCTATCAG	72		amplification of fragments containing both DAB1 and DAB2 loci	
BRD2-R2	CATGAATTCAAGCACAAAGGCCGAGGAGGTGACGA				
DAB1int1-F1	ATCAGAGCGGTGTGGCTATG			sequencicg of DAB1 exon 2	
DAB2int1-F1	GGAGGAGATGAGTGCAAAAG			sequencicg of DAB2 exon 2	

Table S2. Primer pairs used for screening of each positive clones

clone name	positive selection primer pair	negative selection primer pair
Q6A	C3D-S/A	IIBexon4-F1/R1
C3D	IIBexon4-F1/R1	
J15A	IIBexon4-F1/R1	
A11B	IIBexon4-F1/R1	
Q17G	A11B-F/R	A11B BRD2-F/R
K8A	Q13C-S/A	Q13C-F/R
Q13C	TAP1 exon7-F/R	
K6J	Q13C-F/R	Q13C-S/A
T69H	K6J-F/R	MHC I-F/R
O19R	V66E-F/R	
S14A	V66E-F/R	
X65K	V66E-F/R	
O62E	V66E-F/R	
P32B	V66E-F/R	
Z34D	MHC I-F/R	
N49F	Z34D-F/R, MHC I-F/R	
V66E	T69H-F/R	K6J F/R
Z57D	TNXB-S1/A1	
L57C	TNXB-S1/A1	

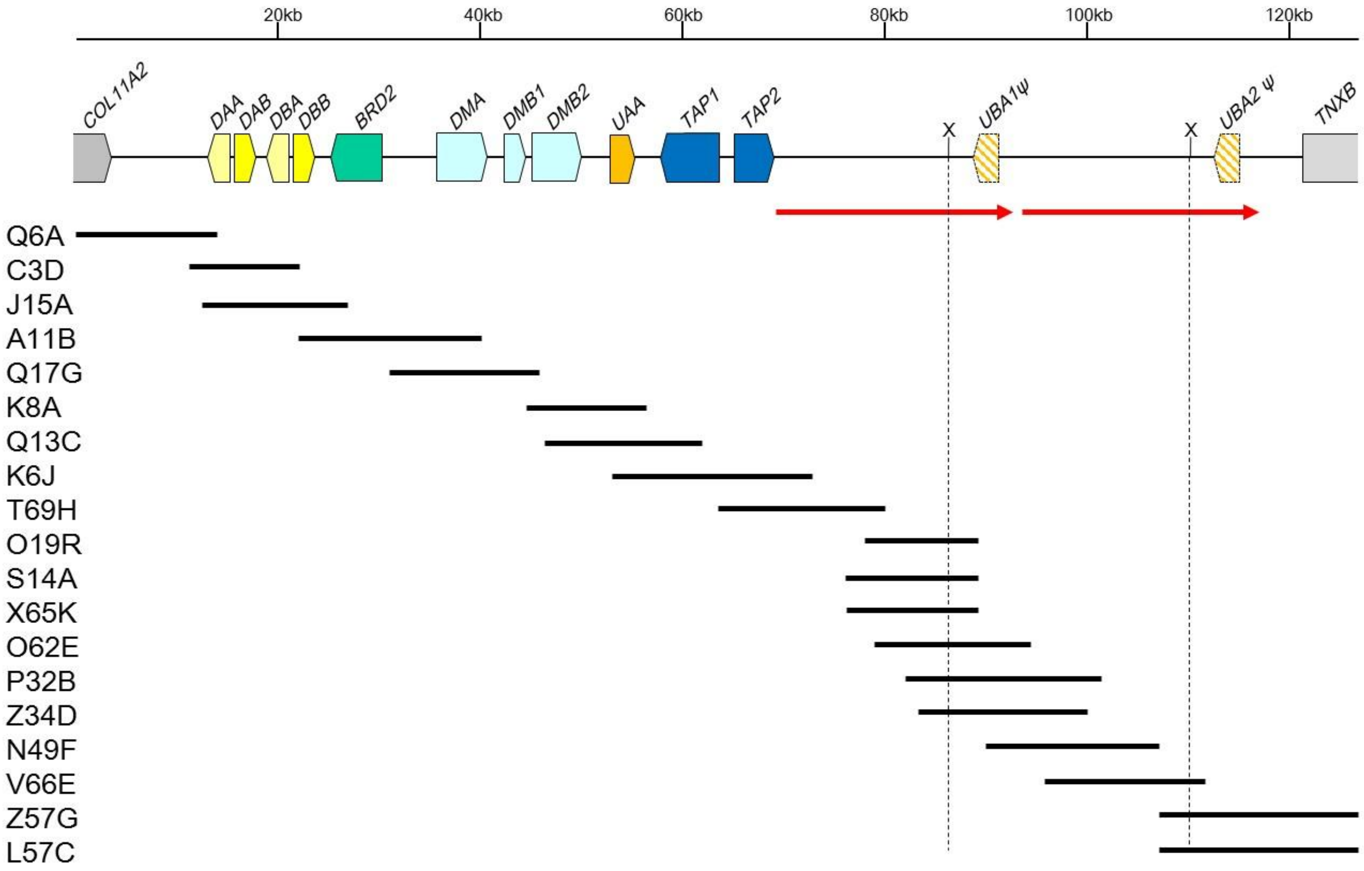

Figure S1. Genomic organization of the Oriental stork MHC. Collagen-type XI α-2 (COL11A2), two copies of MHC IIA/IIB pairs (DAA1/DAB1 and DAA2/DAB2), BRD2, DMA, DMB1, DMB2, MHC I (UAA), TAP1, TAP2, two copies of MHC I (UBA1 and UBA2), and TNXB and their orientations are indicated. All the isolated lambda phage clones are depicted below the map. X on the map indicates the XhoI site used for sequence comparison between the nine phage clones O19R, S14A, X65K, O62E, P32B, Z34D, V66E, Z57G, and L57C (Figure S4).

```
Repeat1     1:GGCCAGAAGCCCCCCCTGGCTCTGGGACACAGGATCCCAAGCACCGTGGGGCTGGGACAGGGCAGCCAGGACCCCCTCGGGATGGCGGACAGGGTGGCTG 100
              ****************************************************************************************************
Repeat2     1:GGCCAGAAGCCCCCCCTGGCTCTGGGACACAGGATCCCAAGCACCGTGGGGCTGGGACAGGGCAGCCAGGACCCCCTCGGGATGGCGGACAGGGTGGCTG 100

Repeat1   101:GGAGCCCTCATGCCCAGTGTCCCTTGTGCCCAGCCTCGCTTGTGCCCGGCGTCCCTCGTGCCTGGCAGCGCTCCCGCCTGGCAGCTCTCGTGCCTGGCAG 200
              ****************************************************************************************************
Repeat2   101:GGAGCCCTCATGCCCAGTGTCCCTTGTGCCCAGCCTCGCTTGTGCCCGGCGTCCCTCGTGCCTGGCAGCGCTCCCGCCTGGCAGCTCTCGTGCCTGGCAG 200

Repeat1   201:TGCTCCTGCCCAGCACCTCCCGTGGCGGGTTGTCCTGGTTTCGGCTGGGATGGAGTTAATTTTCTTCCTCATAGCTGGCATCCTGCTGTGGTTTGGGTTT 300
              ************************************************************************************************ ***
Repeat2   201:TGCTCCTGCCCAGCACCTCCCGTGGCGGGTTGTCCTGGTTTCGGCTGGGATGGAGTTAATTTTCTTCCTCATAGCTGGCATCCTGCTGTGGTTTGGATTT 300

Repeat1   301:CATTGTGCATCACTGCTTTTGTATATTCTTTTAGCATTATTATTATTATTATACTTTTCCCTTCCTTTTCTGTCCTGTTAAACTGTCTTTACCTCAAGCC 400
              ****************************************************************************************************
Repeat2   301:CATTGTGCATCACTGCTTTTGTATATTCTTTTAGCATTATTATTATTATTATACTTTTCCCTTCCTTTTCTGTCCTGTTAAACTGTCTTTACCTCAAGCC 400

Repeat1   401:ATGAATTTTGCGCTTTTCCCCGATTCTCTCCCCCGTCCCGCTAGTTGGGGCGAGTCACCAAACACTGTGGGGGTTAGCTGCCTGCTGGCTGAACCCGCGA 500
              *************************************************************** ************************************
Repeat2   401:ATGAATTTTGCGCTTTTCCCCGATTCTCTCCCCCGTCCCGCTAGTTGGGGCGAGTCACCAAACGCTGTGGGGGTTAGCTGCCTGCTGGCTGAACCCGCGA 500

Repeat1   501:CATCCCCACACGTTCCCACCACGTCCCCAGCTGTGGGGGACATGCAGCACTTGCAGGTGCGTTGTAGTAACACACCTAGACATACAAAGATAGGTTACTA 600
              ******************************************************************************************** *******
Repeat2   501:CATCCCCACACGTTCCCACCACGTCCCCAGCTGTGGGGGACATGCAGCACTTGCAGGTGCGTTGTAGTAACACACCTAGACATACAAAGATATGTTACTA 600

Repeat1   601:ATATTATTGTTCCCAATATTATAAGAAAATACAGCAATAATTCCCTTGGAACACAACCTATATGGGCTATGGATTCTATCTCAGTCCAAGTGCAGTTCAT 700
              ****************************************************************************************************
Repeat2   601:ATATTATTGTTCCCAATATTATAAGAAAATACAGCAATAATTCCCTTGGAACACAACCTATATGGGCTATGGATTCTATCTCAGTCCAAGTGCAGTTCAT 700

Repeat1   701:TTAATTCCGAGTCAGCTTCAATTTCAACGGTGCTGTTTCTCTCGATGTCCGCTCTTCCGGAGGGGAGGTTTTCACCCGTGTGTAGGGAATCCAAGAATCT 800
              ********************************************************************************* ******************
Repeat2   701:TTAATTCCGAGTCAGCTTCAATTTCAACGGTGCTGTTTCTCTCGATGTCCGCTCTTCCGGAGGGGAGGTTTTCACCCGTGTCTAGGGAATCCAAGAATCT 800

Repeat1   801:ATTCCTTTTACTTTGATAGCTGTGGGAGTAATTAGCAATACCAAATATGGTCCCTTCCAATGTTCTTTGAAAGGTTCATCTTTCCAGGTTCGGACGTAGA 900
              ****************************************************************************************************
Repeat2   801:ATTCCTTTTACTTTGATAGCTGTGGGAGTAATTAGCAATACCAAATATGGTCCCTTCCAATGTTCTTTGAAAGGTTCATCTTTCCAGGTTCGGACGTAGA 900

Repeat1   901:CTTGGTCTCCCTGTTGAAAGGAATGTACCGGAGTGTCTAGTGGAACGGGTGTTCTCTCATTCAGGTACCCGTGAAGGGAAGACAAAGTACGCCCCAACGA 1000
              ****************************************************************************************************
Repeat2   901:CTTGGTCTCCCTGTTGAAAGGAATGTACCGGAGTGTCTAGTGGAACGGGTGTTCTCTCATTCAGGTACCCGTGAAGGGAAGACAAAGTACGCCCCAACGA 1000

Repeat1  1001:CATCAGGTATTCAGTCAGTATTTGGTCTCCACTGACATGCGTTTGATCGTGACTCCTATAGGGCGAATTGGTCTTTCCAGGTTCGGACGTAGACTTGGTC 1100
              ************************************************
Repeat2  1001:CATCAGGTATTCAGTCAGTATTTGGTCTCCACTGACATGCGTTTGATC---------------------------------------------------- 1048

Repeat1  1101:TCCCTGTTGAAAGGAATGTACCGGAGTGTCTAGTGGAACGGGTGTTCTCTCATTCAGGTACCCGTGAAGGGAAGACAAAGTACGCCCCAACGACATCAGG 1200

Repeat2  1048:---------------------------------------------------------------------------------------------------- 1048

Repeat1  1201:TATTCAGTCAGTATTTGGTCTCCACTGACATGCGTTTGATCACTCCTGCCTGTTAATTTATTCGCAGGATACAGTTTACCATACAGAATTTCAAAAGGAC 1300
                                                       ***********************************************************
Repeat2  1049:-----------------------------------------ACTCCTGCCTGTTAATTTATTCGCAGGATACAGTTTACCATACAGAATTTCAAAAGGAC 1107

Repeat1  1301:TAAGCCCTTCCCTAACTCTAGGGGTAATCCTGATTCTTAGCAATGCTATAGGTAGACCTTCTATCCATTTCATTTGGCTTTCCTGGCATAATTTTGAAAT 1400
              ****************************************************************************************************
Repeat2  1108:TAAGCCCTTCCCTAACTCTAGGGGTAATCCTGATTCTTAGCAATGCTATAGGTAGACCTTCTATCCATTTCATTTGGCTTTCCTGGCATAATTTTGAAAT 1207

Repeat1  1401:TTGTCTCTTTAAAGTTTGATTCATCCGCTCTACTTTCCCGCTGGACTGCGGTCTCCAAGCGGTCTGGAAATTCCAATCAAACTGTAAAAACTTAGCCACC 1500
              ************** *************************************************************************************
Repeat2  1208:TTGTCTCTTTAAAGCTTGATTCATCCGCTCTACTTTCCCGCTGGACTGCGGTCTCCAAGCGGTCTGGAAATTCCAATCAAACTGTAAAAACTTAGCCACC 1307

Repeat1  1501:TCCTGTACAATTTTAGCAATAAAACGGGGCCCATTATGTGAGGCCATTCCTTCAGGGATGCCGAATCTAGGGGATTATTTCTTTCAACAGTACTCTGACTA 1600
              ****************************************************************************************************
Repeat2  1308:TCCTGTACAATTTTAGCAATAAAACGGGGCCCATTATGTGAGGCCATTCCTTCAGGGATGCCGAATCTAGGGATTATTTCTTTCAACAGTACTCTGACTA 1407

Repeat1  1601:CCTCTCTAGCTTTGTTGGTTCGGCATGGGAAAGCTTCTGGCCATCCCGAAAAGGT--------------------------------------------- 1655
              *******************************************************
Repeat2  1408:CCTCTCTAGCTTTGTTGGTTCGGCATGGGAAAGCTTCTGGCCATCCCGAAAAGGTACCTACTCATACCAATAAGTATTTAATTTGATTACACCTAGGTAA 1507

Repeat1  1655:---------------------------------------------------------------------------------------------------- 1655

Repeat2  1508:CTCAGAGAAATCTGTTTGCCAGTACTCCCCTGGAGTTTGTCCCCCTTTCACCGGTCCCGGAGGAGGTCTCCTCTGTATCTTTGGGTTGTTCTTGGAACAT 1607

Repeat1  1656:------------------------------------------------CCTATGGCATATCGTCTGATGTCTGCTTTTACTGCATCTGCTCCCATATGGG 1707
                                                              ****************************************************
Repeat2  1608:ATCGAGCACTGCTTTACAACAGCATGAGCTAATTTTTGCATTCTTGGTCCTATGGCATATCGTCTGATGTCTGCTTTTACTGCATCTGCTCCCATATGGG 1707
```

```
Repeat1  1708:TTTCTTGATGAGTCCTCTTTATTAATTCCCTCAGTAGTGGGGCAGTGACTATTATTTGTCCAGCATCAGTTGTCCACCATCCGTCCTGGGTTTTGGCACA 1807
              ****************************************************************************************************
Repeat2  1708:TTTCTTGATGAGTCCTCTTTATTAATTCCCTCAGTAGTGGGGCAGTGACTATTATTTGTCCAGCATCAGTTGTCCACCATCCGTCCTGGGTTTTGGCACA 1807

Repeat1  1808:GCTAATTGATTGTCCTTTTCTGAATATGTTGGGGGCTCCAGTAAAATTCTTCCAGTCGGAATTAAAGCCCCCACCTTAGAATCTGTCAAAGCTGCCCTTT 1907
              ***************************************************************                   ******************
Repeat2  1808:GCTAATTGATTGTCCTTTTCTGAATATGTTGGGGGCTCCAGTAAAATTCTTCCAGTCGGAATT-------------------CTGTCAAAGCTGCCCTTT 1888

Repeat1  1908:GTGCTGTCTCGTCAGCTTTTCGATTTCCACTA-ATGATGTCTGTTTGTCCTTTCTAATGGGCTTTACAGTGTATTACAGACGCCTTCCTTGGCTTGAGAA 2006
              ******************************** ********************** ********************************************
Repeat2  1889:GTGCTGTCTCGTCAGCTTTTCGATTTCCACTATATGATGTCTGTTTGTCCTTTCTGATGGGCTTTACAGTGTATTACAGACGCCTTCCTTGGCTTGAGAA 1988

Repeat1  2007:CGGCCTGTAATACCTCTACAATTGCCTTTCCATGTTGAATTGGAGTTCCTTGGGAAGGTAACAGTCCTCTTTCTTTCCGTATGGCTCCATGGGCATGTAC 2106
              ****************************************************************************************************
Repeat2  1989:CGGCCTGTAATACCTCTACAATTGCCTTTCCATGTTGAATTGGAGTTCCTTGGGAAGGTAACAGTCCTCTTTCTTTCCGTATGGCTCCATGGGCATGTAC 2088

Repeat1  2107:CACTCCAAAGGCGTATTTTGAGTCCGAATATGTGTTTACTCTCATATTTTCAGACAATTCCAGCGCTCTGTTTCCTGCAACCATCTCAGCCTTTTGTGCC 2206
              ****************************************************************************************************
Repeat2  2089:CACTCCAAAGGCGTATTTTGAGTCCGAATATGTGTTTACTCTCATATTTTCAGACAATTCCAGCGCTCTGTTTCCTGCAACCATCTCAGCCTTTTGTGCC 2188

Repeat1  2207:GATGTATTAACAGGTCTTTTGCCCTGTGAAACTTCAAAACCTGGGTATTCCACAGTGGCTTTGCCCAATCTGAGCTTTCTTTTGTGATACTCTGTATCCA 2306
              ****************************************************************************************************
Repeat2  2189:GATGTATTAACAGGTCTTTTGCCCTGTGAAACTTCAAAACCTGGGTATTCCACAGTGGCTTTGCCCAATCTGAGCTTTCTTTTGTGATACTCTGTATCCA 2288

Repeat1  2307:GCAAGTCCCCAGAAGTTTAATAGATTGATGGTGGCTCTTTTGCATTCTTGTTCCGAGGCGGTCCCTATGAGTATATCATCTACGTACTGTAGTAAAGTAA 2406
              ****************************************************************************************************
Repeat2  2289:GCAAGTCCCCAGAAGTTTAATAGATTGATGGTGGCTCTTTTGCATTCTTGTTCCGAGGCGGTCCCTATGAGTATATCATCTACGTACTGTAGTAAAGTAA 2388

Repeat1  2407:CAGAAGGATGCTCACCTTGCCAGTCTTCCAGTTCTTTAGCTAGTACGTTACCGAACAAGGTAGGGCTCTTCTTACATCCCTGGGGTAGCACGGTCCAGCA 2506
              ****************************************************************************************************
Repeat2  2389:CAGAAGGATGCTCACCTTGCCAGTCTTCCAGTTCTTTAGCTAGTACGTTACCGAACAAGGTAGGGCTCTTCTTACATCCCTGGGGTAGCACGGTCCAGCA 2488

Repeat1  2507:GAGTTGGACTTTTCTTTCGGTGGCTGGACTTTCCCATTCGAACGCAAACAATTCCTGACTTTCTTCTTCCAGAGGTATACAAAAGAAAGCATCCTTCAAA 2606
              ****************************************************************************************************
Repeat2  2489:GAGTTGGACTTTTCTTTCGGTGGCTGGACTTTCCCATTCGAACGCAAACAATTCCTGACTTTCTTCTTCCAGAGGTATACAAAAGAAAGCATCCTTCAAA 2588

Repeat1  2607:TCCAGTACTGTAAAATAAACATTCTTCTCAGGTATTGATGATAGCAGAGTGTATGGATTTGGAACTACCAGATGCCCATCAGCTGTAATCCAGTTAATTT 2706
              ****************************************************************************************************
Repeat2  2589:TCCAGTACTGTAAAATAAACATTCTTCTCAGGTATTGATGATAGCAGAGTGTATGGATTTGGAACTACCAGATGCCCATCAGCTGTAATCCAGTTAATTT 2688

Repeat1  2707:CTCTTAAATCCTCTACCAACCGATACTCCTGTCTTTGGGGCTTCCTGACCGGCAAAATTGGAGTATTATATTCACACTGGCATTCTCTTAATAGTCCACA 2806
              ****************************************************************************************************
Repeat2  2689:CTCTTAAATCCTCTACCAACCGATACTCCTGTCTTTGGGGCTTCCTGACCGGCAAAATTGGAGTATTATATTCACACTGGCATTCTCTTAATAGTCCACA 2788

Repeat1  2807:CTGAATAAAGTCATTTATCAATGGTTCCAAACCTATTCTAGCTTCCAACTTAATAGGATATTGTCTTTTTCTTACCGGTTGTGCTCCGGGTTTCAATTCA 2906
              ****************************************************************************************************
Repeat2  2789:CTGAATAAAGTCATTTATCAATGGTTCCAAACCTATTCTAGCTTCCAACTTAATAGGATATTGTCTTTTTCTTACCGGTTGTGCTCCGGGTTTCAATTCA 2888

Repeat1  2907:AGTTTTACCGGTACAGCATTTTTTGCTCTTCCCGGTCTCTTGGTTGCCCATACTGTAGGTATGACAACGTCCAGGATTTCCTCTGGGATGTTGACATTCT 3006
              ****************************************************************************************************
Repeat2  2889:AGTTTTACCGGTACAGCATTTTTTGCTCTTCCCGGTCTCTTGGTTGCCCATACTGTAGGTATGACAACGTCCAGGATTTCCTCTGGGATGTTGACATTCT 2988

Repeat1  3007:CCTGTTGCTTTGCAGTCAATAAACAAAATTGTGCTTTCCAGGCAGTTTCCTTTGGTAGACGAAACTGTATTGATCTATCAGAAAAGGTTATTTGTGCTCT 3106
              ****************************************************************************************************
Repeat2  2989:CCTGTTGCTTTGCAGTCAATAAACAAAATTGTGCTTTCCAGGCAGTTTCCTTTGGTAGACGAAACTGTATTGATCTATCAGAAAAGGTTATTTGTGCTCT 3088

Repeat1  3107:TAATTTGCCAAGTAAATCTCGTCCTAGAAGGGGAACGGGGCACTCTGGCATATAGGGAAACTCATGAGATACCACTTTACCTCCAATCTTACATTCCACG 3206
              ****************************************************************************************************
Repeat2  3089:TAATTTGCCAAGTAAATCTCGTCCTAGAAGGGGAACGGGGCACTCTGGCATATAGGGAAACTCATGAGATACCACTTTACCTCCAATCTTACATTCCACG 3188

Repeat1  3207:GGTTTCAGAAATGGTTTCGAAGCTCGCTTTCCCGTGACCCCTACAATCGGCATCTAAATATTACTTAAAGGTCCCTTACAGCTAGTCACTACAGAATGGG 3306
              ****************************************************************************************************
Repeat2  3189:GGTTTCAGAAATGGTTTCGAAGCTCGCTTTCCCGTGACCCCTACAATCGGCATCTAAATATTACTTAAAGGTCCCTTACAGCTAGTCACTACAGAATGGG 3288

Repeat1  3307:TGGCCCCCGTGTGCAAAGAGTTAAAATCCCCAAAGACAAAAAGAAATGTTGTAAATATCTTGGCTGCTCCACTCTAAGGTATGTTTCCTTTTAGTTTTCC 3406
              ****************************************************************************************************
Repeat2  3289:TGGCCCCCGTGTGCAAAGAGTTAAAATCCCCAAAGACAAAAAGAAATGTTGTAAATATCTTGGCTGCTCCACTCTAAGGTATGTTTCCTTTTAGTTTTCC 3388

Repeat1  3407:TTTTGCAGATAATTTAGCCTAGCCTGTCAGTATTGTCGGCCTTAAGTAAGGAAGCTTAGAGAGTAGAATTGCTAACGAGCATTCTTTATTTGAAGGAAAC 3506
              ****************************************************************************************************
Repeat2  3389:TTTTGCAGATAATTTAGCCTAGCCTGTCAGTATTGTCGGCCTTAAGTAAGGAAGCTTAGAGAGTAGAATTGCTAACGAGCATTCTTTATTTGAAGGAAAC 3488

Repeat1  3507:CTACGTATGGTCTTTCCCCCTCCTAACTATAATCATTACAGTAAGAATGCTTTGCCTGTGTTAAATCCTTGGTTAGCTGAGATAGTTGCAATGTAGACAG 3606
              ****************************************************************************************************
Repeat2  3489:CTACGTATGGTCTTTCCCCCTCCTAACTATAATCATTACAGTAAGAATGCTTTGCCTGTGTTAAATCCTTGGTTAGCTGAGATAGTTGCAATGTAGACAG 3588
```

```
Repeat1  3607:AGAAAAAGGAATTCTTCAGGCCCTGTGTCCCTGGAAAGGGCCGATAAGGAGAAGCTACGCAAGAACCAACTGGTTTTGGGTACGCAAGGAAGGTATGAGC 3706
              ****************************************************************************************************
Repeat2  3589:AGAAAAAGGAATTCTTCAGGCCCTGTGTCCCTGGAAAGGGCCGATAAGGAGAAGCTACGCAAGAACCAACTGGTTTTGGGTACGCAAGGAAGGTATGAGC 3688

Repeat1  3707:AAGACAAAGATGGACTGAGCATGCGTAAAGACAAAGTTCAGTCAGCAGCCAGAGCGATGAAGACTACCGACTTCCTCCAACGACCACCAGAGGATCCCTA 3806
              ****************************************************************************************************
Repeat2  3689:AAGACAAAGATGGACTGAGCATGCGTAAAGACAAAGTTCAGTCAGCAGCCAGAGCGATGAAGACTACCGACTTCCTCCAACGACCACCAGAGGATCCCTA 3788

Repeat1  3807:ACGACCACCTAAGGACTTAAGTACGCATGCGTCAGAGACACGTACATATATTAATGAGTTGCAAGAAATCTCATGTTTCTATAAATTAATCTTAGGAATA 3906
              ****************************************************************************************************
Repeat2  3789:ACGACCACCTAAGGACTTAAGTACGCATGCGTCAGAGACACGTACATATATTAATGAGTTGCAAGAAATCTCATGTTTCTATAAATTAATCTTAGGAATA 3888

Repeat1  3907:TGTATGATTTTGCAATACATAATCTCTGCAGTTTTGCTTGTTTGTCGGAGCACTTATGGTGGAGCGATCCCCAGCGCTGCCCAGCGCTGTAATAAAGGAT 4006
              ****************************************************************************************************
Repeat2  3889:TGTATGATTTTGCAATACATAATCTCTGCAGTTTTGCTTGTTTGTCGGAGCACTTATGGTGGAGCGATCCCCAGCGCTGCCCAGCGCTGTAATAAAGGAT 3988

Repeat1  4007:GCCTGCTTCATAGTAACTCCGTTGCTATTGAGTTTTATTATTTCAGGAACACTCTGAAACTCCGATTCACCTGCTGGGAGATTTGGACTTTAAAGGGTAG 4106
              ****************************************************************************************************
Repeat2  3989:GCCTGCTTCATAGTAACTCCGTTGCTATTGAGTTTTATTATTTCAGGAACACTCTGAAACTCCGATTCACCTGCTGGGAGATTTGGACTTTAAAGGGTAG 4088

Repeat1  4107:TCTCCATGAATTGTGCTTCACGTGTCTGTCAGAAAATCTAGAATTTCATTCCCCAGCTGAACTGGAACCAGGGGTCGGCTGGCGAAACTTCAGTGTTCTC 4206
              ****************************************************************************************************
Repeat2  4089:TCTCCATGAATTGTGCTTCACGTGTCTGTCAGAAAATCTAGAATTTCATTCCCCAGCTGAACTGGAACCAGGGGTCGGCTGGCGAAACTTCAGTGTTCTC 4188

Repeat1  4207:CCCGGTCCCCGTCAATCCGAATCTGAGGTGCCCAACATAATTAGATCAGCCTCCCATGTAGATTCCCCAGCCTCGGGTAATCAGTTTTCTCTTCGGGCAT 4306
              ****************************************************************************************************
Repeat2  4189:CCCGGTCCCCGTCAATCCGAATCTGAGGTGCCCAACATAATTAGATCAGCCTCCCATGTAGATTCCCCAGCCTCGGGTAATCAGTTTTCTCTTCGGGCAT 4288

Repeat1  4307:TCATTTTTCCTATGTCCGTCTTCCCTACAGTATGCACACTGGTTCGATCCTAACATGACTTGCAACGGGGACAGACCCAGGGTCGCTACCCTGGCTCCTA 4406
              ****************************************************************************************************
Repeat2  4289:TCATTTTTCCTATGTCCGTCTTCCCTACAGTATGCACACTGGTTCGATCCTAACATGACTTGCAACGGGGACAGACCCAGGGTCGCTACCCTGGCTCCTA 4388

Repeat1  4407:CCTCTGCCTCTGTCATTTTGTGGAGCTACCACCTAGCATCCGGTCTTGCGCAAAACCAGAAATAAATCGGTATCTTGGCTCCATGTGCGTATTGGGTGTT 4506
              ****************************************************************************************************
Repeat2  4389:CCTCTGCCTCTGTCATTTTGTGGAGCTACCACCTAGCATCCGGTCTTGCGCAAAACCAGAAATAAATCGGTATCTTGGCTCCATGTGCGTATTGGGTGTT 4488

Repeat1  4507:GCACACTGGGTAACAAACTCCGTTTGCGAGACAACAATAGCAGACGTACCTTCTCAGCACCCGTCTGCGTGGCCGCAGAGTCGGAGACCTTCTCAACGCC 4606
              ****************************************************************************************************
Repeat2  4489:GCACACTGGGTAACAAACTCCGTTTGCGAGACAACAATAGCAGACGTACCTTCTCAGCACCCGTCTGCGTGGCCGCAGAGTCGGAGACCTTCTCAACGCC 4588

Repeat1  4607:TGTCTGCGTGGCCTCAGAGCAGGCGACCACCTTCTCAGTGCCCCTTTGCAAGGTCTCAGAGCAGGAGACCTGCTGGAGTGCTGGGATGGAGGGCCATCAG 4706
              ****************************************************************************************************
Repeat2  4589:TGTCTGCGTGGCCTCAGAGCAGGCGACCACCTTCTCAGTGCCCCTTTGCAAGGTCTCAGAGCAGGAGACCTGCTGGAGTGCTGGGATGGAGGGCCATCAG 4688

Repeat1  4707:CTCTTCAGGAGGGGTAGGCAGGGCAGGTGAGGTGGAGGAGTTGCCCTGCATAGAAGAGAGAGGTTTGGCCACACAGCCCTTATAGCTGGGGATGACGTGG 4806
              ****************************************************************************************************
Repeat2  4689:CTCTTCAGGAGGGGTAGGCAGGGCAGGTGAGGTGGAGGAGTTGCCCTGCATAGAAGAGAGAGGTTTGGCCACACAGCCCTTATAGCTGGGGATGACGTGG 4788

Repeat1  4807:TCGAGAGCCTCTGGGTGAGGCTTATCACAGAATCGTAGAATGCTTTGGATTGGAAGGAACACCCCTCCTTCTGGCGTGTCAACTGCACCACTCAGCAGCA 4906
              ****************************************************************************************************
Repeat2  4789:TCGAGAGCCTCTGGGTGAGGCTTATCACAGAATCGTAGAATGCTTTGGATTGGAAGGAACACCCCTCCTTCTGGCGTGTCAACTGCACCACTCAGCAGCA 4888

Repeat1  4907:TTAGGGAGATGGAAGGCAAAGCAGATGGCGCAGTGGGTGTCCACTGCCCATCACCCAGCCAGGGTGACAGCACCGATGAGTGATTCCATGGGCAATTAGG 5006
              ****************************************************************************************************
Repeat2  4889:TTAGGGAGATGGAAGGCAAAGCAGATGGCGCAGTGGGTGTCCACTGCCCATCACCCAGCCAGGGTGACAGCACCGATGAGTGATTCCATGGGCAATTAGG 4988

Repeat1  5007:AGAGACCTCTGGATCAGTCGCCCTTGGCCTTACGGGAGACTTCCATGTCCCAGGCATCAGCTGGGAATGCCGTACTGCTGGGGCAAGCAGGTCTGGGAAA 5106
              ****************************************************************************************************
Repeat2  4989:AGAGACCTCTGGATCAGTCGCCCTTGGCCTTACGGGAGACTTCCATGTCCCAGGCATCAGCTGGGAATGCCGTACTGCTGGGGCAAGCAGGTCTGGGAAA 5088

Repeat1  5107:TGCCTGGAGTGTGTGGAAGAGGACTTCTTGTCACAAGCACTCAGCGACCCAACCAGGAGAGATGTCCCTCGAGACTTGTTCTGTGTGAATGGAGAAGGCC 5206
              ****************************************************************************************************
Repeat2  5089:TGCCTGGAGTGTGTGGAAGAGGACTTCTTGTCACAAGCACTCAGCGACCCAACCAGGAGAGATGTCCCTCGAGACTTGTTCTGTGTGAATGGAGAAGGCC 5188

Repeat1  5207:TTGTTGGAGATGTCAGGGTCGGTGGCTGTCTTGGCCACAGGGATCATGAAATGGTTGAGTTTACGGGCGTGCTGGTCAGCAGCAGGCTCAACAGGAGTCA 5306
              ****************************************************************************************************
Repeat2  5189:TTGTTGGAGATGTCAGGGTCGGTGGCTGTCTTGGCCACAGGGATCATGAAATGGTTGAGTTTACGGGCGTGCTGGTCAGCAGCAGGCTCAACAGGAGTCA 5288

Repeat1  5307:GCAGTGTGTCCTGGCAGCCAGGAGGGCAAACTGCATCCTGGGGTGTCCCCATCCCTGGTCCGGGTGCATCAAACACAGCATCATCAGCTGGTCAAGAGAG 5406
              ****************************************************************************************************
Repeat2  5289:GCAGTGTGTCCTGGCAGCCAGGAGGGCAAACTGCATCCTGGGGTGTCCCCATCCCTGGTCCGGGTGCATCAAACACAGCATCATCAGCTGGTCAAGAGAG 5388

Repeat1  5407:GGCATTGTCCCACTGTATTCAGCATCAGTGCAGCTGCTCCTGCAGCACTTTGTGCAGTGCCAGGCCCCACCATTTGAAAAGGACCCAAAGGGCCTTGAAC 5506
              ********************************************** *****************************************************
Repeat2  5389:GGCATTGTCCCACTGTATTCAGCATCAGTGCAGCTGCTCCTGCAGCCCTTTGTGCAGTGCCAGGCCCCACCATTTGAAAAGGACCCAAAGGGCCTTGAAC 5488
```

```
Repeat1  5507:TCCTCCAGAGGAGGGCAACCAAGCTGGTGCCACGGCTGGAAGGCATGTCCTCTGAGGAGCAGCTGAGGACTTTGGGCTTGTCTAGTTTGGAGAGGAGGAG 5606
              ****************************************************************************************************
Repeat2  5489:TCCTCCAGAGGAGGGCAACCAAGCTGGTGCCACGGCTGGAAGGCATGTCCTCTGAGGAGCAGCTGAGGACTTTGGGCTTGTCTAGTTTGGAGAGGAGGAG 5588

Repeat1  5607:ATGCAGACAGGGAGGTGCTGATCTCTTCTCCCTGGGATCCAGGGACAGGACGCCTGGGAATGGTTCAAAGCTGCACCAGGGGAGGCTCAGACTGGACATG 5706
              ****************************************************************************************************
Repeat2  5589:ATGCAGACAGGGAGGTGCTGATCTCTTCTCCCTGGGATCCAGGGACAGGACGCCTGGGAATGGTTCAAAGCTGCACCAGGGGAGGCTCAGACTGGACATG 5688

Repeat1  5707:AGGAAGCATTTCTTTACCAAGAGGCTTCCTGGAGAGGCAGTTGATGCCCCCAGCCTGGCAGGGTTTGAGAGGCATTTGGACAAGGCCCTTAATACACTTG 5806
              ****************************************************************************************************
Repeat2  5689:AGGAAGCATTTCTTTACCAAGAGGCTTCCTGGAGAGGCAGTTGATGCCCCCAGCCTGGCAGGGTTTGAGAGGCATTTGGACAAGGCCCTTAATACACTTG 5788

Repeat1  5807:TGGGCAGCCCTGAAGCAGTCAGGCAGTTGGATGAGATGAGCACTGTAGGCCCCTGCCAGCTAGTCTCTTCTCCTTTCCCAGCACTCAGCTGCATGGCCTC 5906
              ****************************************************************************************************
Repeat2  5789:TGGGCAGCCCTGAAGCAGTCAGGCAGTTGGATGAGATGAGCACTGTAGGCCCCTGCCAGCTAGTCTCTTCTCCTTTCCCAGCACTCAGCTGCATGGCCTC 5888

Repeat1  5907:AGAGCAAGAGAGCTTCTCAGCGCCCATTTGCATGGTGCCAGTGCAAGAGACCTCAGTGACTGTGTTGCACCAGAGGAGGAGACTTTCTAAGGGCCCATCT 6006
              ****** *********************************************************************************************
Repeat2  5889:AGAGCAGGAGAGCTTCTCAGCGCCCATTTGCATGGTGCCAGTGCAAGAGACCTCAGTGACTGTGTTGCACCAGAGGAGGAGACTTTCTAAGGGCCCATCT 5988

Repeat1  6007:GCATGGCCTCAGAGCACCAGACCTTCTTCTCCGTGCACATCTCCTTGACACCAGAGTAGGAGACCTTCTCAGTGTCCATGTGCATGGCCTCAGAGCACAA 6106
              ****************************************************************************************************
Repeat2  5989:GCATGGCCTCAGAGCACCAGACCTTCTTCTCCGTGCACATCTCCTTGACACCAGAGTAGGAGACCTTCTCAGTGTCCATGTGCATGGCCTCAGAGCACAA 6088

Repeat1  6107:GACCTTCTTCTCAGTGCACATCTCCTTGGCACCAGAGCAGGAGACCTTCTCAGTGTCCATCTCCTTGGCACCAGAGCAGGAGACCTTCTCAGTGTCCATG 6206
              ****************************************************************************************************
Repeat2  6089:GACCTTCTTCTCAGTGCACATCTCCTTGGCACCAGAGCAGGAGACCTTCTCAGTGTCCATCTCCTTGGCACCAGAGCAGGAGACCTTCTCAGTGTCCATG 6188

Repeat1  6207:TGCATAGCCTCAGAGCACAAGACCTTCTTCTCCGTGCACATCTCCTTGGCACCAGAGCAGGAGACCTTCTCAGTGTCCATGTGCATAGCCTCAGAGCACA 6306
              ****************************************************************************************************
Repeat2  6189:TGCATAGCCTCAGAGCACAAGACCTTCTTCTCCGTGCACATCTCCTTGGCACCAGAGCAGGAGACCTTCTCAGTGTCCATGTGCATAGCCTCAGAGCACA 6288

Repeat1  6307:AGACCTTCTTCTCCGTGCACATCTCCTTGGCACCAGAGCAGGAGACCTTCTCAGTGTCCATGTGCATGGCCTCAGAGCACAAGACCTTCTTCTC------ 6400
              ******************************************************************* **************************
Repeat2  6289:AGACCTTCTTCTCCGTGCACATCTCCTTGGCACCAGAGCAGGAGACCTTCTCAGTGTCCATGTGCATAGCCTCAGAGCACAAGACCTTCTTCTCCGTGCA 6388

Repeat1  6401:------------------------------------------------------------------------AGCACCCATCGGCATGGCCCCAGAGCAG 6428
                                                                                      ****************************
Repeat2  6389:CATCTTGGCACCAGAGCAGGAGACCTTCTCAGTGTCCATGTGCATGGCCTCAGAGCACAAGACCTTCTTCTCAGCACCCATCGGCATGGCCCCAGAGCAG 6488

Repeat1  6429:GAGAGCTTCTCAGTGCCCCTTTGTGTGGTGCCAGTCCAGAGAGCTCAGTGACCACCGGTGTTGCACCAGAACAGGAGACCTTCTTCTCCGTGCACATCTC 6528
              ****************************************************************************************************
Repeat2  6489:GAGAGCTTCTCAGTGCCCCTTTGTGTGGTGCCAGTCCAGAGAGCTCAGTGACCACCGGTGTTGCACCAGAACAGGAGACCTTCTTCTCCGTGCACATCTC 6588

Repeat1  6529:TGTGGCCTCAGAGCACAAGACCATCTCCCCAGCACCCGTCTGCGTGGCCGCCGAGGAGGACACCATCTCAGCACCCTTCTGGGTGGCTGGGGTGGGTTGA 6628
              ****************************************************************************************************
Repeat2  6589:TGTGGCCTCAGAGCACAAGACCATCTCCCCAGCACCCGTCTGCGTGGCCGCCGAGGAGGACACCATCTCAGCACCCTTCTGGGTGGCTGGGGTGGGTTGA 6688

Repeat1  6629:ACTTTGCTGGCCACCAGGTGCCCAGCCAGCTGCTCTATCATCACTCTACCTACCTCCACTGGCCAAAAAGACTCCACTCCGGGAAATTAATATACTTTAT 6728
              ****************************************************************************************************
Repeat2  6689:ACTTTGCTGGCCACCAGGTGCCCAGCCAGCTGCTCTATCATCACTCTACCTACCTCCACTGGCCAAAAAGACTCCACTCCGGGAAATTAATATACTTTAT 6788

Repeat1  6729:TGCCAATCAAATCACAGCAGGGTAACAAGAAATAAAACCAAACCTTAAAAGCCCTTGCCCCCACCCCTCCCTTCTGCCTGGGCTCACTTTCACACCCAAT 6828
              ****************************************************************************************************
Repeat2  6789:TGCCAATCAAATCACAGCAGGGTAACAAGAAATAAAACCAAACCTTAAAAGCCCTTGCCCCCACCCCTCCCTTCTGCCTGGGCTCACTTTCACACCCAAT 6888

Repeat1  6829:TTCTCTACCTCCTCCCCCCCAGCAGCACAGGGGGAGGGGAATGGGGGTTGCAGTCAGTCCATCACATGTTGTCTCTGCTCCTCCTCCTCCTCACACTCTT 6928
              ****************************************************************************************************
Repeat2  6889:TTCTCTACCTCCTCCCCCCCAGCAGCACAGGGGGAGGGGAATGGGGGTTGCAGTCAGTCCATCACATGTTGTCTCTGCTCCTCCTCCTCCTCACACTCTT 6988

Repeat1  6929:CCTCTGCTCCAGCATGGGGTCCTCTGCAAAATTCTCCACCATGGGTCCTTCAGGGTGCAACCACCTCCTCTGGCAGGGGGTCCTCCAGGGGCTGCAGGTG 7028
              ****************************************************************************************************
Repeat2  6989:CCTCTGCTCCAGCATGGGGTCCTCTGCAAAATTCTCCACCATGGGTCCTTCAGGGTGCAACCACCTCCTCTGGCAGGGGGTCCTCCAGGGGCTGCAGGTG 7088

Repeat1  7029:GGTGTCTGCTCCACCATGGCTCTCCATGGGCTGCCAGGGGACAGCCTGTGTCACCGTGGTCTTCACCACGGGCTGCAGGGGAATCTCTGCTCCGGCACCT 7128
              ****************************************************************************************************
Repeat2  7089:GGTGTCTGCTCCACCATGGCTCTCCATGGGCTGCCAGGGGACAGCCTGTGTCACCGTGGTCTTCACCACGGGCTGCAGGGGAATCTCTGCTCCGGCACCT 7188

Repeat1  7129:GGAGCACCTCCTCGCCTCCTTCTCCACTGACCTTGCTGTCTGCAGAGTTGCCTCTCTCCTGGTGTCTCTCTCCCAGCCGCTGTTGCACAGCAGTTTTTTT 7228
              ****************************************************************************************************
Repeat2  7189:GGAGCACCTCCTCGCCTCCTTCTCCACTGACCTTGCTGTCTGCAGAGTTGCCTCTCTCCTGGTGTCTCTCTCCCAGCCGCTGTTGCACAGCAGTTTTTTT 7288

Repeat1  7229:TCCTCCCCTTCTTAAATACGTGATCCCAGAGGCACTACCACCATCACTGATGGGCTCAGCTTTGGCCAGTTGGCTAATGCCAACCCCGCTCCCCCCCCGC 7328
              ********************************* ******************************************************************
Repeat2  7289:TCCTCCCCTTCTTAAATACGTGATCCCAGAGGCGCTACCACCATCACTGATGGGCTCAGCTTTGGCCAGTTGGCTAATGCCAACCCCGCTCCCCCCCCGC 7388
```

```
Repeat1  7329:TACCAAACCCTTGGTTCCCATCAGAGCTTGGGGCTTGCTGCAGAGATATGGCCGGTTCTGTGAGCAGCTTGAACTCCTGAGGTCACGGTCATTAAGGAAC 7428
              ****************************************************************************************************
Repeat2  7389:TACCAAACCCTTGGTTCCCATCAGAGCTTGGGGCTTGCTGCAGAGATATGGCCGGTTCTGTGAGCAGCTTGAACTCCTGAGGTCACGGTCATTAAGGAAC 7488

Repeat1  7429:AGACAAGGTTCTTGGCCCTGCACCTGCATCCTGCAACAAGAAGGAAAGACACCGCTGGTACCATCGGGTTAGCGGTTGTCCCCTCAAGCACCAGGAGACA 7528
              ****************************************************************************************************
Repeat2  7489:AGACAAGGTTCTTGGCCCTGCACCTGCATCCTGCAACAAGAAGGAAAGACACCGCTGGTACCATCGGGTTAGCGGTTGTCCCCTCAAGCACCAGGAGACA 7588

Repeat1  7529:AGGGCTGCCTGCAAGGGACAGTCCCAGCAAGACATGGCCCACCAGGCCCCTGCCCATCCCACTGCAGGACCGTCACCCCACGGCGCCGGCTTGCAATGGG 7628
              ****************************************************************************************************
Repeat2  7589:AGGGCTGCCTGCAAGGGACAGTCCCAGCAAGACATGGCCCACCAGGCCCCTGCCCATCCCACTGCAGGACCGTCACCCCACGGCGCCGGCTTGCAATGGG 7688

Repeat1  7629:ACTCACGTGTCATTGAAGCTGCTGCCGTCCACCCACTCCAAGTGCTCGCCCTGTCTCCGCAGCCCAAGCCAGTAATCAATGTTGCCCTTGAGGCGCAAGA 7728
              ****************************************************************************************************
Repeat2  7689:ACTCACGTGTCATTGAAGCTGCTGCCGTCCACCCACTCCAAGTGCTCGCCCTGTCTCCGCAGCCCAAGCCAGTAATCAATGTTGCCCTTGAGGCGCAAGA 7788

Repeat1  7729:GAAACTCCTGGGGAGGAAAGAGAGAAGCCAAGAGTCAGGAGCTGCTGCCAGCCCCACGCTGGTCCCTGCTGGCCTCGCAGGCAGCCCCAGCTCCGCACCG 7828
              ****************************************************************************************************
Repeat2  7789:GAAACTCCTGGGGAGGAAAGAGAGAAGCCAAGAGTCAGGAGCTGCTGCCAGCCCCACGCTGGTCCCTGCTGGCCTCGCAGGCAGCCCCAGCTCCGCACCG 7888

Repeat1  7829:CTGCCACCGGCCACGGGATGGCAGCAGCTCCCACCCAACGGCTGCTCCAACAAGCTCCAGAGACTTCTGAAGGAGCTCACCAGCCCAGCCAGCAGTGTTG 7928
              ****************************************************************************************************
Repeat2  7889:CTGCCACCGGCCACGGGATGGCAGCAGCTCCCACCCAACGGCTGCTCCAACAAGCTCCAGAGACTTCTGAAGGAGCTCACCAGCCCAGCCAGCAGTGTTG 7988

Repeat1  7929:CTCTGCTCTGCCAGAGCTCTGCTCGGGGCCCAGCTGAGCACCGCGGGTGGGCAGACATAGCCTGGGCTCCCCGCTGCACCCCACGCCTCCCTCCCTCCCT 8028
              ****************************************************************************************************
Repeat2  7989:CTCTGCTCTGCCAGAGCTCTGCTCGGGGCCCAGCTGAGCACCGCGGGTGGGCAGACATAGCCTGGGCTCCCCGCTGCACCCCACGCCTCCCTCCCTCCCT 8088

Repeat1  8029:CCAAGGCCCCAGGGCAGAGCTGCATCTCTGTCCCTACAGGCACCTCTCGCCTGCCCAGCACCAACCCCAGCACCTGCACCCAACCCAGACCCCCCCCGCC 8128
              ****************************************************************************************************
Repeat2  8089:CCAAGGCCCCAGGGCAGAGCTGCATCTCTGTCCCTACAGGCACCTCTCGCCTGCCCAGCACCAACCCCAGCACCTGCACCCAACCCAGACCCCCCCCGCC 8188

Repeat1  8129:TTTCACCCCCACGCAGCCACAACAGCCCCTCACTCACCTTTTCCCAGTCCCTCTTGAACACAGCCAGGGAGGACCCACGCAAGGGAGCACTGCTTGCGGC 8228
              ************************************************************************************ ***************
Repeat2  8189:TTTCACCCCCACGCAGCCACAACAGCCCCTCACTCACCTTTTCCCAGTCCCTCTTGAACACAGCCAGGGAGGACCCACGCAAGG-AGCACTGCTTGCGGC 8287

Repeat1  8229:TCCACTCCCAGCTGCCCTCCTCCCTCGAGAGGTAGTAGCAGACATTGCGGTACCCAACCCAGTCGTCAGGACACGGCAGCACCTGAGCCACAGGCAGACC 8328
              ****************************************************************************************************
Repeat2  8288:TCCACTCCCAGCTGCCCTCCTCCCTCGAGAGGTAGTAGCAGACATTGCGGTACCCAACCCAGTCGTCAGGACACGGCAGCACCTGAGCCACAGGCAGACC 8387

Repeat1  8329:TGCATTGCCTTCACACCTTCCTGCTGGAGAAGGAAGGGGAGAAGGTCAGAGGATTCCACTCCACAGGGACCAGGGGACACAGCTCCAGGGGGCAGGAAAG 8428
              ****************************************************************************************************
Repeat2  8388:TGCATTGCCTTCACACCTTCCTGCTGGAGAAGGAAGGGGAGAAGGTCAGAGGATTCCACTCCACAGGGACCAGGGGACACAGCTCCAGGGGGCAGGAAAG 8487

Repeat1  8429:GAAACAGTCCCAGGCTCCCCCCACAGGGATCCTCACTTTGCTGCCAGGGACCTACAGGGAGCAGACACCCCGGGGGTTAACAGCCTGCCCCACAGCACGG 8528
              ****************************************************************************************************
Repeat2  8488:GAAACAGTCCCAGGCTCCCCCCACAGGGATCCTCACTTTGCTGCCAGGGACCTACAGGGAGCAGACACCCCGGGGGTTAACAGCCTGCCCCACAGCACGG 8587

Repeat1  8529:CTGGGGGTGTCCCTGCTCCTCCCCAAGACCCCTGGGACAGGGCAGGGGCACAGCCAGCCTGAGGGCCTGGCCCCACCATGCTCCCCCCACCAGCCTGGGC 8628
              ****************************************************************************************************
Repeat2  8588:CTGGGGGTGTCCCTGCTCCTCCCCAAGACCCCTGGGACAGGGCAGGGGCACAGCCAGCCTGAGGGCCTGGCCCCACCATGCTCCCCCCACCAGCCTGGGC 8687

Repeat1  8629:CTCACACACCCACATCTGCCCCAGCCTGGAGGGGAAGGGGCCGCATCTGCAAGGGCAGCTGCTCCTCCCTTACCTGATACTACAGCAAGAGCTACAGCCA 8728
              ****************************************************************************************************
Repeat2  8688:CTCACACACCCACATCTGCCCCAGCCTGGAGGGGAAGGGGCCGCATCTGCAAGGGCAGCTGCTCCTCCCTTACCTGATACTACAGCAAGAGCTACAGCCA 8787

Repeat1  8729:AAGCCAACACCAGAACCATGAGCACAGCCAGCACCAGGGCCCATGCTGGATGGAGTGCACAGCATTTGCCTGGAGAAGGAAAAAGCCACTCACAGTGAGG 8828
              ****************************************************************************************************
Repeat2  8788:AAGCCAACACCAGAACCATGAGCACAGCCAGCACCAGGGCCCATGCTGGATGGAGTGCACAGCATTTGCCTGGAGAAGGAAAAAGCCACTCACAGTGAGG 8887

Repeat1  8829:ATGGCGCTGTCCCAGAGCGGGCACAGCCCTCGCATCCCTGCCACCCCGCACACGAGCTGCCAGGGGAACACAGGAGGGACCCCCCCAAGGGACAGCCTGC 8928
              ****************************************************************************************************
Repeat2  8888:ATGGCGCTGTCCCAGAGCGGGCACAGCCCTCGCATCCCTGCCACCCCGCACACGAGCTGCCAGGGGAACACAGGAGGGACCCCCCCAAGGGACAGCCTGC 8987

Repeat1  8929:AGCAGGAGAGCTGCCGACGGATGGAGATGTGGTGGGGGGCTGCTACAGACCACCCAATGAGGACCAAGCAGAAGACAAGCCCTTTCCAGACAACTGGAAG 9028
              ****************************************************************************************************
Repeat2  8988:AGCAGGAGAGCTGCCGACGGATGGAGATGTGGTGGGGGGCTGCTACAGACCACCCAATGAGGACCAAGCAGAAGACAAGCCCTTTCCAGACAACTGGAAG 9087

Repeat1  9029:AAGCCTCACACTTGCCAGCCCCAGTCCTCCTGGGGTGACACCGCCACAGGGACACTATCCCCTCCCTACAGGCCACAGGGCTGGCTCTGCACTCACCCAG 9128
              ****************************************************************************************************
Repeat2  9088:AAGCCTCACACTTGCCAGCCCCAGTCCTCCTGGGGTGACACCGCCACAGGGACACTATCCCCTCCCTACAGGCCACAGGGCTGGCTCTGCACTCACCCAG 9187

Repeat1  9129:AACTCTGCGTGTCCTTACGCCTTTATCCCCAGGCACCCCTGGACCCAGGGGCTCCTGCACGTTCCCAGCGTCATCGCAGAACCCGTTCTCCTCCCCTCGT 9228
              ****************************************************************************************************
Repeat2  9188:AACTCTGCGTGTCCTTACGCCTTTATCCCCAGGCACCCCTGGACCCAGGGGCTCCTGCACGTTCCCAGCGTCATCGCAGAACCCGTTCTCCTCCCCTCGT 9287
```

```
Repeat1  9229:CCCATGGGAAACAGAGACATGGAATCACTTTGCCTGTGATAAGCAGGGGCTTGCGCCCAAGGCATCGCAACAGCTGGCAAAGCCTTTTTTAAGCCAGGCT 9328
              ****************************************************************************************************
Repeat2  9288:CCCATGGGAAACAGAGACATGGAATCACTTTGCCTGTGATAAGCAGGGGCTTGCGCCCAAGGCATCGCAACAGCTGGCAAAGCCTTTTTTAAGCCAGGCT 9387

Repeat1  9329:TCGATCCAGTTTTACCACAGCAGCTGGTACTGTCGTGCTGGGAGACTGCAGAGAACATGATGAGAACACAGCACACCGGGACGTTTAATTCCGTTACCAG 9428
              ******************************************************************* ********************************
Repeat2  9388:TCGATCCAGTTTTACCACAGCAGCTGGTACTGTCGTGCTGGGAGACTGCAGAGAACATGATGAGAACGCAGCACACCGGGACGTTTAATTCCGTTACCAG 9487

Repeat1  9429:ACTGCAGCCACGCTTGGCTTCCGCAGATCAGCTGTGGCCCTGCGGCAACACCCACGCTCGGGAAGGCGACGCCAGCACAGTCCCGCTCTGTCTGTGCTGC 9528
              ****************************************************************************************************
Repeat2  9488:ACTGCAGCCACGCTTGGCTTCCGCAGATCAGCTGTGGCCCTGCGGCAACACCCACGCTCGGGAAGGCGACGCCAGCACAGTCCCGCTCTGTCTGTGCTGC 9587

Repeat1  9529:CCCTGTAACGCTGCAGCCTGTCCCCAGCACCCAGCCAGCACCCGGGGAGACAAACGCAAGGTCAGTGAGCTCAGCCCCCAGCAGAGCCAGCACCCTCCCC 9628
              ********************************           *********************************************************
Repeat2  9588:CCCTGTAACGCTGCAGCCTGTCCCCAGCACCC-----------GGGGAGACAAACGCAAGGTCAGTGAGCTCAGCCCCCAGCAGAGCCAGCACCCTCCCC 9676

Repeat1  9629:ACCACCCTGCACACACCCTTCCCAGACAGGCTACAAGGCACTCGCTGCCTGGGAGCATCCCCCTAACCACCTCTGCGGACACACAGGAAGGAAGTGCCAG 9728
              ****************************************************************************************************
Repeat2  9677:ACCACCCTGCACACACCCTTCCCAGACAGGCTACAAGGCACTCGCTGCCTGGGAGCATCCCCCTAACCACCTCTGCGGACACACAGGAAGGAAGTGCCAG 9776

Repeat1  9729:TAACCACAGAGGAAGGCAGGAACTGGGTGCGCCTGGCAGGACAAGGCACCCCAGAAGCCCCCAGACACCCAGACTAGAAGCTCAGCATATCCTTAACAGA 9828
              ****************************************************************************************************
Repeat2  9777:TAACCACAGAGGAAGGCAGGAACTGGGTGCGCCTGGCAGGACAAGGCACCCCAGAAGCCCCCAGACACCCAGACTAGAAGCTCAGCATATCCTTAACAGA 9876

Repeat1  9829:CAAATCCTTAACAGGCCCAGACATGTCAAGGACACCGAGTCAAAGTCACTGCAAAACGCCCCGCTCCCATCTGAACGCAGGATACCGAGGTTTGGTCTTG 9928
              ****************************************************************************************************
Repeat2  9877:CAAATCCTTAACAGGCCCAGACATGTCAAGGACACCGAGTCAAAGTCACTGCAAAACGCCCCGCTCCCATCTGAACGCAGGATACCGAGGTTTGGTCTTG 9976

Repeat1  9929:CCTGGGTTTTACAAATGCATGAGCTCACAGGGTTAAAGGGGGACAGGAGGGCAGGGCTGCTCTCCTGCAGCCCCTGGGCATCCCAGAGCCTGCTCAGCTG 10028
              ****************************************************************************************************
Repeat2  9977:CCTGGGTTTTACAAATGCATGAGCTCACAGGGTTAAAGGGGGACAGGAGGGCAGGGCTGCTCTCCTGCAGCCCCTGGGCATCCCAGAGCCTGCTCAGCTG 10076

Repeat1 10029:GCAGAGCTCCAGCTGGTAGCTGGGCACCCACAACTTTTAGTAGGGCCCGTAGCAATAGGACAAGGCACAATGGTTTAAGGTAGAAAAGGCCAGATTCAGA 10128
              ****************************************************************************************************
Repeat2 10077:GCAGAGCTCCAGCTGGTAGCTGGGCACCCACAACTTTTAGTAGGGCCCGTAGCAATAGGACAAGGCACAATGGTTTAAGGTAGAAAAGGCCAGATTCAGA 10176

Repeat1 10129:CCAGGTCCAAGGAAGAAATTCGAAATTTTTTACGCTGAGGGTGGTGAGACACGGGCCCAGGCTGCTCACAGAGATGGGACATGCCCCATCCCTGGGAACG 10228
              ****************************************************************************************************
Repeat2 10177:CCAGGTCCAAGGAAGAAATTCGAAATTTTTTACGCTGAGGGTGGTGAGACACGGGCCCAGGCTGCTCACAGAGATGGGACATGCCCCATCCCTGGGAACG 10276

Repeat1 10229:TTCAAGGTCGGCTTGGACGGGGCTCTGAGGAACCTGCTCTAGCTGAAGAAGTCCCTGCCCGGGGCAGGGGGGCTGGACTAAATCAGCTTTAAGCGCCCCT 10328
              ****************************************************************************************************
Repeat2 10277:TTCAAGGTCGGCTTGGACGGGGCTCTGAGGAACCTGCTCTAGCTGAAGAAGTCCCTGCCCGGGGCAGGGGGGCTGGACTAAATCAGCTTTAAGCGCCCCT 10376

Repeat1 10329:TCCACCCCAAACCACTCCATGAGTCTGTGATCACAGACACCGACGCCTACGGGGGTACGGCTGGGGCCCCTCAGCAGCTCCCGCACCGCCGAGCTGAACG 10428
              ****************************************************************************************************
Repeat2 10377:TCCACCCCAAACCACTCCATGAGTCTGTGATCACAGACACCGACGCCTACGGGGGTACGGCTGGGGCCCCTCAGCAGCTCCCGCACCGCCGAGCTGAACG 10476

Repeat1 10429:ACGGCGAGCTGAAGGAAAGCCTCCGCAGGGGCCCTCCTCTCCTACACGATCCACAAGCGGCTGCAGCTGCTCGGGCACAGGCACATCCCAGCGCCCGGGA 10528
              ****************************************************************************************************
Repeat2 10477:ACGGCGAGCTGAAGGAAAGCCTCCGCAGGGGCCCTCCTCTCCTACACGATCCACAAGCGGCTGCAGCTGCTCGGGCACAGGCACATCCCAGCGCCCGGGA 10576

Repeat1 10529:ACTGCCGGCTGCGGGCACAGGACACGGACAGCACCGGCTGAGCCCCCGCAAGGGATCACGCCAGTCACCCCAGGGGCGCTGGGGGGCGGGGGGAACCCGC 10628
              ****************************************************************************************************
Repeat2 10577:ACTGCCGGCTGCGGGCACAGGACACGGACAGCACCGGCTGAGCCCCCGCAAGGGATCACGCCAGTCACCCCAGGGGCGCTGGGGGGCGGGGGGAACCCGC 10676

Repeat1 10629:GCGAAGCAGCTCCCGCCACGCCGGCGCCTCACAGCGTTCCCAACCCCGGCACGGCTCAGGGCACAGCGGCAAGGGGGACGCCACAACCGGCCGCTCCCGC 10728
              ****************************************************************************************************
Repeat2 10677:GCGAAGCAGCTCCCGCCACGCCGGCGCCTCACAGCGTTCCCAACCCCGGCACGGCTCAGGGCACAGCGGCAAGGGGGACGCCACAACCGGCCGCTCCCGC 10776

Repeat1 10729:ACCGGCCCCACCACGCGGCCAGCGCCAGCCCGACAGGCTCCCTTCCCCGCGGACCCCCACGCACCCGCCGCCCGCGCCCTACCTGCGCCGGGGCCCGCCC 10828
              ****************************************************************************************************
Repeat2 10777:ACCGGCCCCACCACGCGGCCAGCGCCAGCCCGACAGGCTCCCTTCCCCGCGGACCCCCACGCACCCGCCGCCCGCGCCCTACCTGCGCCGGGGCCCGCCC 10876

Repeat1 10829:CGAGCGCTGCCGCCGCCGCCCCGCCGGGATCCGGACGCTCCCCGGCCCCGCACGGCTCTGACAGGAAACGCTCGCGGCCGCCGCCTTTCGAGGCCTCGCG 10928
              ****************************************************************************************************
Repeat2 10877:CGAGCGCTGCCGCCGCCGCCCCGCCGGGATCCGGACGCTCCCCGGCCCCGCACGGCTCTGACAGGAAACGCTCGCGGCCGCCGCCTTTCGAGGCCTCGCG 10976

Repeat1 10929:CGGCCCGCCCACCGCGCTGCCCAATCAGCGCGCGAGAGCGAGGAGTGGCGGGGCAGGCAGCCAATCAGGCGACGCGCCTTTGTAACGCCCTCTGCGTGAG 11028
              ****************************************************************************************************
Repeat2 10977:CGGCCCGCCCACCGCGCTGCCCAATCAGCGCGCGAGAGCGAGGAGTGGCGGGGCAGGCAGCCAATCAGGCGACGCGCCTTTGTAACGCCCTCTGCGTGAG 11076

Repeat1 11029:CGGGACGCGGTGCTGCCCTATCCTTCTGCCCAGGCGCTCTAGTTTGGCCGGAACCGTGGTCGTTTTGCGCCCGCACCGACATCGGCGGGCGCAGGACACC 11128
              ****************************************************************************************************
Repeat2 11077:CGGGACGCGGTGCTGCCCTATCCTTCTGCCCAGGCGCTCTAGTTTGGCCGGAACCGTGGTCGTTTTGCGCCCGCACCGACATCGGCGGGCGCAGGACACC 11176
```

Repeat1 11129:CCCAATTCCAGCAGCCGAGGCTGCTGCCATGGTTGCGGGCCGTTTCCCTGCTCGTGCGGGGCGAGCAGCCCCTGGCAATGCCCGCTCCCGGCTCCCTCAG 11228
****
Repeat2 11177:CCCAATTCCAGCAGCCGAGGCTGCTGCCATGGTTGCGGGCCGTTTCCCTGCTCGTGCGGGGCGAGCAGCCCCTGGCAATGCCCGCTCCCGGCTCCCTCAG 11276

Repeat1 11229:GCGGTTTCCCCCATTTTTGGGGAGCGAGGGGCGCTGGGGGGGGTTTGAGATGGAAACCCCCTTGCCTCACCAGTGTATCAGTCAGCTTTAGAGTATAAAA 11328
****
Repeat2 11277:GCGGTTTCCCCCATTTTTGGGGAGCGAGGGGCGCTGGGGGGGGTTTGAGATGGAAACCCCCTTGCCTCACCAGTGTATCAGTCAGCTTTAGAGTATAAAA 11376

Repeat1 11329:GCGTATTAACTGGGTTAATACGCGTTATTTCACACCATACGCTGCGATGCAATGGGGTAGTGGAATTTTCCTGGAGGTTTTGATATGAAATAAAGCAAAT 11428
****
Repeat2 11377:GCGTATTAACTGGGTTAATACGCGTTATTTCACACCATACGCTGCGATGCAATGGGGTAGTGGAATTTTCCTGGAGGTTTTGATATGAAATAAAGCAAAT 11476

Repeat1 11429:TAAGGACGGGCCGGCACCTGGAAAGAAGGACTTTGCTTCTCAGGAGCTTGGAGCTGCCCGTGGAGATAAGGGATCGCCCTGGACAACGCCGATGCTGCCA 11528
****
Repeat2 11477:TAAGGACGGGCCGGCACCTGGAAAGAAGGACTTTGCTTCTCAGGAGCTTGGAGCTGCCCGTGGAGATAAGGGATCGCCCTGGACAACGCCGATGCTGCCA 11576

Repeat1 11529:GCATGTGCCCCTCCGCCACCTCCTTGAAACCCTCCCGGCATCATGGATAACTGATTCCAGCAAGACCGAGCCCGGGGACGTCGGCATCAATACGGTGCAA 11628
****
Repeat2 11577:GCATGTGCCCCTCCGCCACCTCCTTGAAACCCTCCCGGCATCATGGATAACTGATTCCAGCAAGACCGAGCCCGGGGACGTCGGCATCAATACGGTGCAA 11676

Repeat1 11629:TGAATGCTGAAAAGCAGTCGCTTTGCGATGTTTTATGGGGATTAGTAATCGTTGGTGTTGGTGTTAGTCGTCGAGGTGTCGCGGGTTAGTGGCAGGAGTC 11728
****
Repeat2 11677:TGAATGCTGAAAAGCAGTCGCTTTGCGATGTTTTATGGGGATTAGTAATCGTTGGTGTTGGTGTTAGTCGTCGAGGTGTCGCGGGTTAGTGGCAGGAGTC 11776

Repeat1 11729:CCAGGACTGGAGTGGTGGGACGGAGGGCCATCGACTCTTCAGGAGGGCAGGCGAGGGATGTAAGGGAGAGGTTTGGCTGCACAGCCCTTACAGTTGGGGA 11828
****
Repeat2 11777:CCAGGACTGGAGTGGTGGGACGGAGGGCCATCGACTCTTCAGGAGGGCAGGCGAGGGATGTAAGGGAGAGGTTTGGCTGCACAGCCCTTACAGTTGGGGA 11876

Repeat1 11829:TGACATGGTCGAGAGCCTGCGGGTGAGGATTAGGGGGATGGAAGACAAAGCAGATGGCACAGTGGGTGTCCACTACCAGCCAGGTGACCAGCCTGGTGCC 11928
****************************** ***
Repeat2 11877:TGACATGGTCGAGAGCCTGCGGGTGAGGATCAGGGGGATGGAAGACAAAGCAGATGGCACAGTGGGTGTCCACTACCAGCCAGGTGACCAGCCTGGTGCC 11976

Repeat1 11929:CAGGCAGGGTGACAGCACCAACGAGAATTAGGAGAAATCTGTGGATCGGTCACCGTTGGCCTTATGGGAGACTTCAAGGTCCCAGGCATCGGCTGGGAAT 12028
** *****************************
Repeat2 11977:CAGGCAGGGTGACAGCACCAACGAGAATTAGGAGAAATCTGTGGATCGGTCACCGTTGGCCTTATGGGAGGCTTCAAGGTCCCAGGCATCGGCTGGGAAT 12076

Repeat1 12029:GCTGTACTGCTGGGACGAGCAGGTCTGGGAAATGCCTGGAGTGTGTGGAAGATGACTTCTTGTCACAAGTCCTCAGTGATACCCACCCTGCAGCCCGTGG 12128
****
Repeat2 12077:GCTGTACTGCTGGGACGAGCAGGTCTGGGAAATGCCTGGAGTGTGTGGAAGATGACTTCTTGTCACAAGTCCTCAGTGATACCCACCCTGCAGCCCGTGG 12176

Repeat1 12129:TGAAGACCATGGTGAGGCAGGCTTTCCCCCTGCAGCCCATGGAGGTGCACGGTGGAGCAGATATCTACCTCCAGCCTGTGGAGTACCCCATGCCGGAGCA 12228
****
Repeat2 12177:TGAAGACCATGGTGAGGCAGGCTTTCCCCCTGCAGCCCATGGAGGTGCACGGTGGAGCAGATATCTACCTCCAGCCTGTGGAGTACCCCATGCCGGAGCA 12276

Repeat1 12229:GGTGGATGCCCGAAGGAGGCTGTGAGCCCGTGGGAAGCCTGTGCTGGAGCAGGCTCCTGGCAGGACCTGTGGGCCCATGGAGAGAGGAGCCCACACTGGA 12328
****
Repeat2 12277:GGTGGATGCCCGAAGGAGGCTGTGAGCCCGTGGGAAGCCTGTGCTGGAGCAGGCTCCTGGCAGGACCTGTGGGCCCATGGAGAGAGGAGCCCACACTGGA 12376

Repeat1 12329:ACAGGTTTGCTGGCAGGACTTGTGACCCCGCGGGGCACCCACGCTGGAGCAGTTCGTGAAGAACTGCAGCCCATGGAAGGACCCACGTTGGAGAAGTTCA 12428
****
Repeat2 12377:ACAGGTTTGCTGGCAGGACTTGTGACCCCGCGGGGCACCCACGCTGGAGCAGTTCGTGAAGAACTGCAGCCCATGGAAGGACCCACGTTGGAGAAGTTCA 12476

Repeat1 12429:CGGAGAACTGTCTCCCGTGGGAGGGAGCCCACGCTGGAGCAGTGGAAGAGCGTGAGGAGGAAGGAGCAGCAGAGACAACGTGTGATGAACTGACCACAAC 12528
****
Repeat2 12477:CGGAGAACTGTCTCCCGTGGGAGGGAGCCCACGCTGGAGCAGTGGAAGAGCGTGAGGAGGAAGGAGCAGCAGAGACAACGTGTGATGAACTGACCACAAC 12576

Repeat1 12529:CCCCATTCCCTGTCCCCCTGCAGCGCTGGGGGGAAGGAGGTAGAGAAATCAGGCATGGAGTTGAGCCCGGAAGAAGGGAGGGGTGGGGGAAGGTGTTTTA 12628
****
Repeat2 12577:CCCCATTCCCTGTCCCCCTGCAGCGCTGGGGGGAAGGAGGTAGAGAAATCAGGCATGGAGTTGAGCCCGGAAGAAGGGAGGGGTGGGGGAAGGTGTTTTA 12676

Repeat1 12629:AGGTTTGGTTTTATTTCTCGTTACCCTACTCTGATTTGATTGGCAATGAATTATATTAATTTCCCTGATTTGAGTCTGTTTTGCCCGTGACGGTAGTTGG 12728
****
Repeat2 12677:AGGTTTGGTTTTATTTCTCGTTACCCTACTCTGATTTGATTGGCAATGAATTATATTAATTTCCCTGATTTGAGTCTGTTTTGCCCGTGACGGTAGTTGG 12776

Repeat1 12729:TGAGCGATCTCCCTGTCCTTATCTTGACCCATGATCCTTTTGTGGTATTTTTCTCCCCCCTGTCCTGTTGAGGAGGGGGAGCGATAGAGCGGCTGGGTGG 12828
****
Repeat2 12777:TGAGCGATCTCCCTGTCCTTATCTTGACCCATGATCCTTTTGTGGTATTTTTCTCCCCCCTGTCCTGTTGAGGAGGGGGAGCGATAGAGCGGCTGGGTGG 12876

Repeat1 12829:GCACCTGGCAGCCAGCCAGCGTCAACCCACCACAGTCAACTAGGAAAGGTGTCCTAGACTTGTTGTTTGTGGATAGAAGTATAGAATCGTTGAGGTTGGA 12928
****
Repeat2 12877:GCACCTGGCAGCCAGCCAGCGTCAACCCACCACAGTCAACTAGGAAAGGTGTCCTAGACTTGTTGTTTGTGGATAGAAGTATAGAATCGTTGAGGTTGGA 12976

Repeat1 12929:AAAGACCTTTAAGAACACCGAGTCCAACTGTAAACCCAACGCTGCCAAGCCCACCACTACACCATGTCCCTAAGCACCACATCTACACGTCTTTTAAATA 13028
****
Repeat2 12977:AAAGACCTTTAAGAACACCGAGTCCAACTGTAAACCCAACGCTGCCAAGCCCACCACTACACCATGTCCCTAAGCACCACATCTACACGTCTTTTAAATA 13076

```
Repeat1 13029:CTTCCAGGGGTGGTGACTCAACCCCTTCCCTGCGCAGCCTGTTCCAATGCTTGACAACGCTTTCAGTGAGGAAATCTTCCCTAATATCCAATCCAAACCT 13128
              ******************************** *******************************************************************
Repeat2 13077:CTTCCAGGGGTGGTGACTCAACCCCTTCCCTGTGCAGCCTGTTCCAATGCTTGACAACGCTTTCAGTGAGGAAATCTTCCCTAATATCCAATCCAAACCT 13176

Repeat1 13129:GCCCTGGCGCAACTCTTGTCCTATCACTTGTTACTTGGGAGAAGAGATCGACCCCCACCTTGCTACAAGCTCCTTTCAGGTAGTTGTAGAGAGCGATAAG 13228
              ****************************************************************************************************
Repeat2 13177:GCCCTGGCGCAACTCTTGTCCTATCACTTGTTACTTGGGAGAAGAGATCGACCCCCACCTTGCTACAAGCTCCTTTCAGGTAGTTGTAGAGAGCGATAAG 13276

Repeat1 13229:GTCTGCCCTGAGCCTCCTTTTCTCCAGACTAAACAAGCCCAGTTCCCTCAGCCGCTCCTCATCAGACTTGTGCTCCAGACCCTTCCCCAGCTCCGTTGCC 13328
              ****************************************************************************************************
Repeat2 13277:GTCTGCCCTGAGCCTCCTTTTCTCCAGACTAAACAAGCCCAGTTCCCTCAGCCGCTCCTCATCAGACTTGTGCTCCAGACCCTTCCCCAGCTCCGTTGCC 13376

Repeat1 13329:CTTCTCTGGACATGCTCCAGCCCCTCAGTGTCTCTCTTGTAGTGAGGGGCCCAAAACTGACCACGGTATTGGAGGTGCGGCCTCTCCAGTGCCGAGTGCA 13428
              *************************** ************************************************************************
Repeat2 13377:CTTCTCTGGACATGCTCCAGCCCCTCAATGTCTCTCTTGTAGTGAGGGGCCCAAAACTGACCACGGTATTGGAGGTGCGGCCTCTCCAGTGCCGAGTGCA 13476

Repeat1 13429:GGGGGACGGTCACTTCCCTAGTCCTGCTGGCCACCCTATTTCTGACACAAGCCAGGATGCTGTTGGCCTTCACAGTATCGTTTAGGCGGGAATTAGAGAA 13528
              ****************************************************************************************************
Repeat2 13477:GGGGGACGGTCACTTCCCTAGTCCTGCTGGCCACCCTATTTCTGACACAAGCCAGGATGCTGTTGGCCTTCACAGTATCGTTTAGGCGGGAATTAGAGAA 13576

Repeat1 13529:GGACTCGTGGGAGAGGTGATGGTCGGTGGCTGTCTTGGCCACAGGGATCGTGAAATGGTTGAGTTTACGGGGGTGCTGGTCGGCAGCAGGATCAATAGGA 13628
              ****************************************************************************************************
Repeat2 13577:GGACTCGTGGGAGAGGTGATGGTCGGTGGCTGTCTTGGCCACAGGGATCGTGAAATGGTTGAGTTTACGGGGGTGCTGGTCGGCAGCAGGATCAATAGGA 13676

Repeat1 13629:GCCAGCAGTGTGTCCTGGCAGCCAAGAGGGCAAACAGCATCCTGGGGTGCATCAAACGTGGCGCAACCAGCTGGTCAAGAGAGGGGATTCTAGAATATCT 13728
              ****************************************************************************************************
Repeat2 13677:GCCAGCAGTGTGTCCTGGCAGCCAAGAGGGCAAACAGCATCCTGGGGTGCATCAAACGTGGCGCAACCAGCTGGTCAAGAGAGGGGATTCTAGAATATCT 13776

Repeat1 13729:GGAGTTGGAAGGGACCCATAAGGATCATGGAGTCCAACTCCCTGCTCCTTGCAGGACTACCTAACACTCATGTGGCTCGTCCTTAATGTGACATATTTGG 13828
              ****************************************************************************************************
Repeat2 13777:GGAGTTGGAAGGGACCCATAAGGATCATGGAGTCCAACTCCCTGCTCCTTGCAGGACTACCTAACACTCATGTGGCTCGTCCTTAATGTGACATATTTGG 13876

Repeat1 13829:CGTCACGATTTTACTTCCCCCTCCCCACAGGTGGGACGTGACACCATCCACCCAGACCCCCAGATCTCTTTCCGCGGGGCTGCTCTCCAGCCTCTCGTCC 13928
              ****************************************************************************************************
Repeat2 13877:CGTCACGATTTTACTTCCCCCTCCCCACAGGTGGGACGTGACACCATCCACCCAGACCCCCAGATCTCTTTCCGCGGGGCTGCTCTCCAGCCTCTCGTCC 13976

Repeat1 13929:CCCAATTTATACGTATAACCAGGATTACCGTGTCCCAGGTGCAGAATCCGGCTCTTGCTCTTGATAAATTCCAGACGGTTCATGATTGCCCAGCTCTGCA 14028
              ****************************************************************************************************
Repeat2 13977:CCCAATTTATACGTATAACCAGGATTACCGTGTCCCAGGTGCAGAATCCGGCTCTTGCTCTTGATAAATTCCAGACGGTTCATGATTGCCCAGCTCTGCA 14076

Repeat1 14029:GTGTGCCCAGATCTCTCTGAAAACCCTCTCCCCTCCCCTCTCCTTCCACATTCCATGCTCCTGTGTCCTCCCTGCCGTGTCCCTGTGTCCCCCGCTGTGT 14128
              ****************************************************************************************************
Repeat2 14077:GTGTGCCCAGATCTCTCTGAAAACCCTCTCCCCTCCCCTCTCCTTCCACATTCCATGCTCCTGTGTCCTCCCTGCCGTGTCCCTGTGTCCCCCGCTGTGT 14176

Repeat1 14129:CCCCGTATCCTCTCTGCCCTCCCACTTCGCCCACCCCCACGTCCCTGTGTTCTCCATGTCCCCGCACCCTCCTGGCTGTGTCCCCATGCCCATTGCCCAT 14228
              *************************** ************************************************************************
Repeat2 14177:CCCCGTATCCTCTCTGCCCTCCCACTTTGCCCACCCCCACGTCCCTGTGTTCTCCATGTCCCCGCACCCTCCTGGCTGTGTCCCCATGCCCATTGCCCAT 14276

Repeat1 14229:GTTCCCATGTTGTCCTGGACACATCCCTGTCTCTGTCTTTTGTGTCCCCAGGTCCTCCCCTCCATGTCCCCATGCCTTGCCATCTCCCCATGGCCTCTCT 14328
              ************************************************************************************************* **
Repeat2 14277:GTTCCCATGTTGTCCTGGACACATCCCTGTCTCTGTCTTTTGTGTCCCCAGGTCCTCCCCTCCATGTCCCCATGCCTTGCCATCTCCCCATGGCCTCCCT 14376

Repeat1 14329:TGCCACATCCCTGTGTCCCCTGCTGTGTCCCCGTATCCTCCCTGCCACATCCCCACACTGGTCCCACGATGTCCCTGTGCCCTCCCACATCCCCCCACCA 14428
              ****************************************************************************************************
Repeat2 14377:TGCCACATCCCTGTGTCCCCTGCTGTGTCCCCGTATCCTCCCTGCCACATCCCCACACTGGTCCCACGATGTCCCTGTGCCCTCCCACATCCCCCCACCA 14476

Repeat1 14429:TGTTCCTGTGTCCTCCATGTCGCCACACCTTCCCAGCAGTGTCCCCATGCCCGTTCCCCATATCCCCACATCCTCCTGGATGCATCCCCATGCCCGTTCC 14528
              ********************* ******************************************************************************
Repeat2 14477:TGTTCCTGTGTCCTCCATGTCCCCACACCTTCCCAGCAGTGTCCCCATGCCCGTTCCCCATATCCCCACATCCTCCTGGATGCATCCCCATGCCCGTTCC 14576

Repeat1 14529:CCATGTCGCCACACCCTCCCCGCCATGTCCCCTCTGTGTCCCAGGTCCTGCTGCGCTACTAGCCCAACATGACCAAGGCCGTGGGCTCCTCAGAGAAGAT 14628
              ****************************************************************************************************
Repeat2 14577:CCATGTCGCCACACCCTCCCCGCCATGTCCCCTCTGTGTCCCAGGTCCTGCTGCGCTACTAGCCCAACATGACCAAGGCCGTGGGCTCCTCAGAGAAGAT 14676

Repeat1 14629:CTTTGAGTTCCTAGACCAGGAGGAGCAGGTGGTACCCCCGGGGACGCTGGCGCCCGATGTCCTGTGGGGCCACGTCCAGCTTGAGGATGTCTGGTTCTCC 14728
              ************************************* **************************************************************
Repeat2 14677:CTTTGAGTTCCTAGACCAGGAGGAGCAGGTGGTACCCACGGGGACGCTGGCGCCCGATGTCCTGTGGGGCCACGTCCAGCTTGAGGATGTCTGGTTCTCC 14776

Repeat1 14729:TACCCTGAGCACCAAGAGCCCATCCTCAAGGTGGGCATGGGGACAGGGCAGGGGATGCGAGGACATGATGGGGGGAGACGGTGGGGAGGGCATGGGGACA 14828
              ********************************** *****************************************************************
Repeat2 14777:TACCCTGAGCACCAAGAGCCCATCCTCAAGGTGGTCATGGGGACAGGGCAGGGGATGCGAGGACATGATGGGGGGAGACGGTGGGGAGGGCATGGGGACA 14876

Repeat1 14829:TGGTGGGGACATGCCAGGTGGACATGGTGGGGAAGGCATGGGGACATGGACGTGGTGGGAGGGTGTGGGGACATAGCAGGGGGCCATGGGGCCATGGCAT 14928
              ****************************************************************************************************
Repeat2 14877:TGGTGGGGACATGCCAGGTGGACATGGTGGGGAAGGCATGGGGACATGGACGTGGTGGGAGGGTGTGGGGACATAGCAGGGGGCCATGGGGCCATGGCAT 14976
```

```
Repeat1 14929:GGGGACATAGAGGTGCTGTGAAGGGCGTTGGGACGTGGCATGGGGACACAGACTTGGTGGGGGGGTGTGGGGACATGGGGGTGGACAGAGGGACATGGTG 15028
              ****************************************************************************************************
Repeat2 14977:GGGGACATAGAGGTGCTGTGAAGGGCGTTGGGACGTGGCATGGGGACACAGACTTGGTGGGGGGGTGTGGGGACATGGGGGTGGACAGAGGGACATGGTG 15076

Repeat1 15029:GGGACATGGCAGGTGGACACGGGGACGTGGCATGGGGAGACAGGTGTGGTGGGGAGGGTGTAGGGACATGGCGGGGGGCCATGGGGGCATGGTAGGAACA 15128
              ****************************************************************************************************
Repeat2 15077:GGGACATGGCAGGTGGACACGGGGACGTGGCATGGGGAGACAGGTGTGGTGGGGAGGGTGTAGGGACATGGCGGGGGGCCATGGGGGCATGGTAGGAACA 15176

Repeat1 15129:TGGTGGGGAGAGCATGGGGAGGAGTGGGGACCCTGGGGGAGGGGGCATGGGGGTGTCACATCTGTCCCCCCAGGGCATGGCCCTGGAGCTGTGCCCCAGG 15228
              ****************************************************************************************************
Repeat2 15177:TGGTGGGGAGAGCATGGGGAGGAGTGGGGACCCTGGGGGAGGGGGCATGGGGGTGTCACATCTGTCCCCCCAGGGCATGGCCCTGGAGCTGTGCCCCAGG 15276

Repeat1 15229:GAGGTGCTGGCGGTGGTGGATCCCCCCGGGACGGGGAAGAGCACCCTGGTGTCCCTGGTCCTGCACCTGCACCCGCCGGGGACCAGGCGGATGCTGCTG 15328
              ******************************** *******************************************************************
Repeat2 15277:GAGGTGCTGGCGGTGGTGGATCCCCCCGGGACAGGGAAGAGCACCCTGGTGTCCCTGGTCCTGCACCTGCACCCGCCGGGGACCAGGCGGATGCTGCTG 15376

Repeat1 15329:GATGGTCACCCCCTCTCTGCCTATGAGAGCGCCCCTACCTGCGCTGCCAGGTGTCACCACCGCCCCTGTCACACGACGTCCCCAATGGGTCCCGACTGCC 15428
              ****************************************************************************************************
Repeat2 15377:GATGGTCACCCCCTCTCTGCCTATGAGAGCGCCCCTACCTGCGCTGCCAGGTGTCACCACCGCCCCTGTCACACGACGTCCCCAATGGGTCCCGACTGCC 15476

Repeat1 15429:ACCCCCATGTCACCAGCCATCCCCACCATGGCCTCGCCACCACCCCGTGTCACCAGACATCCCTGCTGCGTCCCCAGACATCCCTGCCATGTCCACGGGC 15528
              ****************************************************************************************************
Repeat2 15477:ACCCCCATGTCACCAGCCATCCCCACCATGGCCTCGCCACCACCCCGTGTCACCAGACATCCCTGCTGCGTCCCCAGACATCCCTGCCATGTCCACGGGC 15576

Repeat1 15529:CATCCCCACCATGTCCCCACCACTTCTCCCACCACGTCCTCTGCCATGTCCCCAGCCGTCCCCACCACGTCCCCAATGCATCCCTGCCATATCCCCAGCT 15628
              ****************************************************************************************************
Repeat2 15577:CATCCCCACCATGTCCCCACCACTTCTCCCACCACGTCCTCTGCCATGTCCCCAGCCGTCCCCACCACGTCCCCAATGCATCCCTGCCATATCCCCAGCT 15676

Repeat1 15629:GTGTCCCCAGCCATCCCTGCCACATCCCTGCCACGTCCCCTGCCACATCCCCGCTGAGTCCCCAGCCATCCCCACCATGTCCCTGCCATGTCCCCAGCTA 15728
              ****************************************************************************************************
Repeat2 15677:GTGTCCCCAGCCATCCCTGCCACATCCCTGCCACGTCCCCTGCCACATCCCCGCTGAGTCCCCAGCCATCCCCACCATGTCCCTGCCATGTCCCCAGCTA 15776

Repeat1 15729:TCCATGCCATGTCCCTGACGTGTCCCCTGCCACGTTCCCACTGAGTCCCCAGCCATCCCTGCCACGTCCCTGCAGTGTCCCTGCCATGTCCCTGCAGTGT 15828
              ************************************************************************************** *************
Repeat2 15777:TCCATGCCATGTCCCTGACGTGTCCCCTGCCACGTTCCCACTGAGTCCCCAGCCATCCCTGCCACGTCCCTGCAGTGTCCCTGCCACGTCCCTGCAGTGT 15876

Repeat1 15829:ACCTGCTACATCCTTGTCACATCCCCCACCACATCCCCACCATGTCTCCACTGTGTCCCCAGCCACATCCCAGCCATGTCCCTGGAGCATCCTCTGCCAC 15928
              ****************************************************************************************************
Repeat2 15877:ACCTGCTACATCCTTGTCACATCCCCCACCACATCCCCACCATGTCTCCACTGTGTCCCCAGCCACATCCCAGCCATGTCCCTGGAGCATCCTCTGCCAC 15976

Repeat1 15929:ATCCTCGGCACGTTCCCTGCCAAGTCCCCATCGAGTCCCTACGTGAGCCTGCTGTGTCCCTGCAGTGTCCCCTGCCACATCCTCCTCATGTCCTCCACCA 16028
              ******************************** *******************************************************************
Repeat2 15977:ATCCTCGGCACGTTCCCTGCCAAGTCCCCATCAAGTCCCTACGTGAGCCTGCTGTGTCCCTGCAGTGTCCCCTGCCACATCCTCCTCATGTCCTCCACCA 16076

Repeat1 16029:TGTCTGCACCGAGTCCCCAGCCATCCCTGCCATGTCTCTGCTATGTCCCCGCCACGTCCACTGCCATCCCTGTCATATCCCCGCCATGTCCCCAGCATGT 16128
              ****************************************************************************************************
Repeat2 16077:TGTCTGCACCGAGTCCCCAGCCATCCCTGCCATGTCTCTGCTATGTCCCCGCCACGTCCACTGCCATCCCTGTCATATCCCCGCCATGTCCCCAGCATGT 16176

Repeat1 16129:CCCCACCACATCCCCTGCCACATCCCCACCATGTCCCTGCCATGTCCCCTGCCATGTCCCCACTGTGTCCCCACCCATCCCAGCCACATCCTCACCATGT 16228
              ************************************************************************************************ ***
Repeat2 16177:CCCCACCACATCCCCTGCCACATCCCCACCATGTCCCTGCCATGTCCCCTGCCATGTCCCCACTGTGTCCCCACCCATCCCAGCCACATCCTCACCGTGT 16276

Repeat1 16229:CCCCACCCGGTCCCCAGCCACATCCCCTGCCACTTCCCCACCATGTCCCCGCTATGTCCCCAGCCACATCCCAGCCATGTCCCTGCCCTTTCCCCTACCA 16328
              *********************************************** ****************************************************
Repeat2 16277:CCCCACCCGGTCCCCAGCCACATCCCCTGCCACTTCCCCACCATGTCTCCGCTATGTCCCCAGCCACATCCCAGCCATGTCCCTGCCCTTTCCCCTACCA 16376

Repeat1 16329:CATCCCCACTGTGTCCCCAGCCACACCCTTGCCATGTCCCCTGCCACATCCCCGCTGTGTCCCCAGCCACACCCTTGCCATGTCCCCTGCCACGTTCCCG 16428
              ******************************************************************************* ********************
Repeat2 16377:CATCCCCACTGTGTCCCCAGCCACACCCTTGCCATGTCCCCTGCCACATCCCCGCTGTGTCCCCAGCCACACCCTTGCCGTGTCCCCTGCCACGTTCCCG 16476

Repeat1 16429:CTGGGTCCCCAGCCATCCCCGTCATGTCCTTGCCACACCCCCACCATGTCCCCGCGGCATCGCCAGCCACATCCCTGCCACGTCCCTGCCACATCCTCGC 16528
              ************************************************** *************************************************
Repeat2 16477:CTGGGTCCCCAGCCATCCCCGTCATGTCCTTGCCACACCCCCACCATGTCTCCGCGGCATCGCCAGCCACATCCCTGCCACGTCCCTGCCACATCCTCGC 16576

Repeat1 16529:CGAGTCCCCTGCCAACCCTGCCACATCCCCACCATGTCCCCACTGTGTCCCCAGCCATCCCCTCCGCCACAGCTGGCTGCCATCCCCCAGGAGCTGGTGC 16628
               ***************************************************************************************************
Repeat2 16577:TGAGTCCCCTGCCAACCCTGCCACATCCCCACCATGTCCCCACTGTGTCCCCAGCCATCCCCTCCGCCACAGCTGGCTGCCATCCCCCAGGAGCTGGTGC 16676

Repeat1 16629:TCTTCTCCCGCTCACTCCATGCCAACATTGCGTGGCAACCATGCAACATTGGCAGGGGGTGGCCATCGCTCGTGCCCTGGTGAGGGACCCCTGTGTCCTC 16728
              *******  *******************************************************************************************
Repeat2 16677:TCTTCTCTTGCTCACTCCATGCCAACATTGCGTGGCAACCATGCAACATTGGCAGGGGGTGGCCATCGCTCGTGCCCTGGTGAGGGACCCCTGTGTCCTC 16776

Repeat1 16729:ATCCTTGATGAGCCCACGAGCGCCCTGCATGCCGAGAGCCAGCTGCAGGTGGGAGGTGACACCCGTGTCCCCTCGGTCCCCCTGTTCAGCAGCACCCTGG 16828
              ****************************************************************************************************
Repeat2 16777:ATCCTTGATGAGCCCACGAGCGCCCTGCATGCCGAGAGCCAGCTGCAGGTGGGAGGTGACACCCGTGTCCCCTCGGTCCCCCTGTTCAGCAGCACCCTGG 16876
```

```
Repeat1 16829:TCCCTGGGGGTCCCCGGGTGCCCTCTTTTTGTCGTCCCCTTGATCCCCAATTTCGGTGTCCCCTGGGTTGCTGGCCCCTGTGCCCATGTGGTCCCCACT 16928
              ***************************************************************************** **********************
Repeat2 16877:TCCCTGGGGGTCCCCGGGTGCCCTCTTTTTGTCGTCCCCTTGATCCCCAATTTCGGTGTCCCCTGGGTTGCTGGCCGCTGTGCCCATGTGGTCCCCACT 16976

Repeat1 16929:TCCATGTCCCCTTGGTCCCTGGTCCCTGTGTCCCCCTGGGTCCCTGGTGCCCAGGTCCCCTTGCCCCCAGTCCTTCTGTTCCCTGGTCCCTGTGGCTCCT 17028
              ************************************************************************** *************************
Repeat2 16977:TCCATGTCCCCTTGGTCCCTGGTCCCTGTGTCCCCCTGGGTCCCTGGTGCCCAGGTCCCCTTGCCCCCAGTCCTGCTGTTCCCTGGTCCCTGTGGCTCCT 17076

Repeat1 17029:GGGATCCTCTCCCAGCAGCCCCCTAGTGCTGGGGGGGTCCCCCCTGGATCCCCTCTCCTGTGTCCCCTGGGTCTCTGGTCCCCATGTCACCTTGGTCCTT 17128
              ****************************************************************************************************
Repeat2 17077:GGGATCCTCTCCCAGCAGCCCCCTAGTGCTGGGGGGGTCCCCCCTGGATCCCCTCTCCTGTGTCCCCTGGGTCTCTGGTCCCCATGTCACCTTGGTCCTT 17176

Repeat1 17129:GGTCCCTGGTCCCCTTGGTCCCAACTCCCCATGTCCCCTGGGTCCCTGCTCCCCGTGTCCCCTTGGTCCTTGGGCCCCATGTCCCCTGGGTCCCCACAGC 17228
              ****************************************************************************************************
Repeat2 17177:GGTCCCTGGTCCCCTTGGTCCCAACTCCCCATGTCCCCTGGGTCCCTGCTCCCCGTGTCCCCTTGGTCCTTGGGCCCCATGTCCCCTGGGTCCCCACAGC 17276

Repeat1 17229:CAGGGGTCCCCACTGACAATGCCACCCACCACAGGTGGAGCAGGAGATCTTCAGAGCCAGCGGGATGGGGCGCGCAGTGCTGCTGGTGACGGTGCAGGTG 17328
              ****************************************************************************************************
Repeat2 17277:CAGGGGTCCCCACTGACAATGCCACCCACCACAGGTGGAGCAGGAGATCTTCAGAGCCAGCGGGATGGGGCGCGCAGTGCTGCTGGTGACGGTGCAGGTG 17376

Repeat1 17329:GCCCTGGCCATGCGGGTGCTGCGGGTGGCCGTGCTGGAGGGGGGGACGACTGTACGAGCTGGGGTCCCCTGGGGAGCTCCTGCGCCCTGGCAGCCACTACT 17428
              ************ ****** ********************************************************************************
Repeat2 17377:GCCCTGGCCATGTGGGTGCCGCGGGTGGCCGTGCTGGAGGGGGGGACGACTGTACGAGCTGGGGTCCCCTGGGGAGCTCCTGCGCCCTGGCAGCCACTACT 17476

Repeat1 17429:GGCACCAGCTGCAG-------------------------------------------------------------------------------------- 17442
              **************
Repeat2 17477:GGCACCAGCTGCAGCCTCTCCTGTGTCCCCTGGGTCTCTGGTCCCCATGTCACCTTGGTCCTTGGTCCCTGGTCCCCTTGGTCCCAACTCCCCATGTCCC 17576

Repeat1 17442:---------------------------------------------------------------------------------------------------- 17442

Repeat2 17577:CTGGGTCCCTGCTCCCCGTGTCCCCTTGGTCCTTGGGCCCCATGTCCCCTGGGTCCCCACAGCCAGGGGTCCCCACTGACAATGCCACCCACCACAGGTG 17676

Repeat1 17442:---------------------------------------------------------------------------------------------------- 17442

Repeat2 17677:GAGCAGGAGATCTTCAGAGCCAGCGGGATGGGGCGCGCAGTGCTGCTGGTGACGGTGCAGGTGGCCCTGGCCATGTGGGTGCCGCGGGTGGCCGTGCTGG 17776

Repeat1 17443:-----------------------------------------------------------------------------GGCAGGGGAGGGGGGTGGAAGGT 17465
                                                                                           *** *******************
Repeat2 17777:AGGGGGGGACGACTGTACGAGCTGGGGTCCCCTGGGGAGCTCCTGCGCCCTGGCAGCCACTACTGGCACCAGCTGCAGGGCTGGGGAGGGGGGTGGAAGGT 17876

Repeat1 17466:GGGGG------ACACGGGGAAGGAGAGTGAGGGACAGCAGGAGCTGGGGATGGGGACACCGGGAGCCAGCATGGGGACACCAAGAGATGGGATAGGGCCA 17559
              *****      *****************************************************************************************
Repeat2 17877:GGGGGATGGGGACACGGGGAAGGAGAGTGAGGGACAGCAGGAGCTGGGGATGGGGACACCGGGAGCCAGCATGGGGACACCAAGAGATGGGATAGGGCCA 17976

Repeat1 17560:CCAGGAGCAGGGATGGGACACCGAAAGTTGTGATAAGGGACCGTGAGAGCTGTGATGGGGGAGCATGGGCGCCAGGCCAGACATGCTGCCACGTCCCTGC 17659
              ****************************************************************************************************
Repeat2 17977:CCAGGAGCAGGGATGGGACACCGAAAGTTGTGATAAGGGACCGTGAGAGCTGTGATGGGGGAGCATGGGCGCCAGGCCAGACATGCTGCCACGTCCCTGC 18076

Repeat1 17660:TGCGTGATGGTGACCACGGGGGCCAGCAGGTGACACTGGGTACCAGCGGGGCTGGTAACTGGCAGGGCTGGCCAATGGGACACAGAGCTTGAAATAAAGG 17759
              ****************************************************************************************************
Repeat2 18077:TGCGTGATGGTGACCACGGGGGCCAGCAGGTGACACTGGGTACCAGCGGGGCTGGTAACTGGCAGGGCTGGCCAATGGGACACAGAGCTTGAAATAAAGG 18176

Repeat1 17760:CTGTTTGTACCCAGGATGCGTTGGGGGACCCTGGAGCTGGGAAGGGTAAGGGGCTGTAGGGATGGGGGG---------------------------GTGC 17832
              ****** **************************************************************                           ****
Repeat2 18177:CTGTTTATACCCAGGATGCGTTGGGGGACCCTGGAGCTGGGAAGGGTAAGGGGCTGTAGGGATGGGGGGGTGCAGGGATGGGGGGCTGAATTAGGGGTGC 18276

Repeat1 17833:AGGGATGGGGGGTGCAGGGTTTGGGGCAGCAATGGTGTGGGCAGGGGGGCCCCTCTGCCCCATTGGGACAGAAATTTGTACAAAATAAAGCAGAATTGCT 17932
              ****************************************************************************************************
Repeat2 18277:AGGGATGGGGGGTGCAGGGTTTGGGGCAGCAATGGTGTGGGCAGGGGGGCCCCTCTGCCCCATTGGGACAGAAATTTGTACAAAATAAAGCAGAATTGCT 18376

Repeat1 17933:TTGGAGAGGAACTGGCTGGGTTGCGGGGAGGTGGGGGGAACAGTCTGGCCTGGCTGCGGGGTCTTGGGCGCTCACCCTCCAGTTATCACTGCTGCCTTGG 18032
              ****************************************************************************************************
Repeat2 18377:TTGGAGAGGAACTGGCTGGGTTGCGGGGAGGTGGGGGGAACAGTCTGGCCTGGCTGCGGGGTCTTGGGCGCTCACCCTCCAGTTATCACTGCTGCCTTGG 18476

Repeat1 18033:TGTGACGCGGCACTTGCCTCGGTGTGATATAATTAATCTGCCGGCCTCCCCCGCGTGATATCGCAGCCACCGCCCTTTCTACCCGAGAGGCTTCACTCGC 18132
              ******************* ******************************** ***********************************************
Repeat2 18477:TGTGACGCGGCACTTGCCTTGGTGTGATATAATTAATCTGCCGGCCTCCCCCTCGTGATATCGCAGCCACCGCCCTTTCTACCCGAGAGGCTTCACTCGC 18576

Repeat1 18133:TTGATTCCGGCACATCGTTCGCGTCAATGAATGACCTGTGGGATGGCTGCAGTGGTGAGGTCCCCCCCTCCATCGTGGGCCCACCCGTGCATTGTGTCTC 18232
              ****************************************************************************************************
Repeat2 18577:TTGATTCCGGCACATCGTTCGCGTCAATGAATGACCTGTGGGATGGCTGCAGTGGTGAGGTCCCCCCCTCCATCGTGGGCCCACCCGTGCATTGTGTCTC 18676

Repeat1 18233:TCCCCCGGCGTTACACGGGCCCATCGAGCTGTGAACAGCTCACAACAACATGCCCAGTCCAGGTCTACCTGAACCAACAATTGTTGCCGCCTGGTCTGCC 18332
              ************************************************************************************************* **
Repeat2 18677:TCCCCCGGCGTTACACGGGCCCATCGAGCTGTGAACAGCTCACAACAACATGCCCAGTCCAGGTCTACCTGAACCAACAATTGTTGCCGCCTGGTCTACC 18776
```

```
Repeat1 18333:TGCTCCGTGCGTTGATGTTCTCCAGGGGCATGGCTCTTGGACATGGGCACGATGGACCTTTACACCCAGGGGCTCTCCCCAGGCAGCAAGGTCCTGCTGC 18432
              *****************************************  *********************************************************
Repeat2 18777:TGCTCCGTGCGTTGATGTTCTCCAGGGGCATGGCTCTTGGATGTGGGCACGATGGACCTTTACACCCAGGGGCTCTCCCCAGGCAGCAAGGTCCTGCTGC 18876

Repeat1 18433:CATAACCCAGCAGCCCAGAGGGGCTACCTCTGTGACCATTGCTGTAGCCACCCCCACGGGGCACTGGCCACCCTCGAGGAGTCAGTCCAGAGGCAGAGCA 18532
              ********************** ************************************************ ****************************
Repeat2 18877:CATAACCCAGCAGCCCAGAGGGTCTACCTCTGTGACCATTGCTGTAGCCACCCCCACGGGGCACTGGCCACTCTCGAGGAGTCAGTCCAGAGGCAGAGCA 18976

Repeat1 18533:CTGGCCACATTTCTCAGGCAGCAATCTCTAGGGCCAGCTGGGTGGCTTGGACTTGGGCAGCTTCCTCGAAGGACGAAAGATGGAGAGAGACTTTATACCA 18632
              ****************************************************************************************************
Repeat2 18977:CTGGCCACATTTCTCAGGCAGCAATCTCTAGGGCCAGCTGGGTGGCTTGGACTTGGGCAGCTTCCTCGAAGGACGAAAGATGGAGAGAGACTTTATACCA 19076

Repeat1 18633:AGGGCTGTAGTGACAGGACAAGGGCCGTGGAAACGGCAAGAGGGGAGATCGAGATTGGACGTAAGAAATGTTTTAGGATGAGGATGGTGAGAGGCTGGAA 18732
              ****************************************************************************************************
Repeat2 19077:AGGGCTGTAGTGACAGGACAAGGGCCGTGGAAACGGCAAGAGGGGAGATCGAGATTGGACGTAAGAAATGTTTTAGGATGAGGATGGTGAGAGGCTGGAA 19176

Repeat1 18733:CAGGTTGCGCGGAGAAGGTGTGGAAGCTCCATCATTGGAAGTGTTTGAGGTGAGAGCAGACGAGAGGAAACCGCCTCAAGTTGCGCCAGGGGACTTGCTG 18832
              ********** *****************************************************************************************
Repeat2 19177:CAGGTTGCGCAGAGAAGGTGTGGAAGCTCCATCATTGGAAGTGTTTGAGGTGAGAGCAGACGAGAGGAAACCGCCTCAAGTTGCGCCAGGGGACTTGCTG 19276

Repeat1 18833:TGTCTCCTGTCACACTGCTGTGTGATACATCTCCCTATCTACAGCGGCCCACTGGTGCCCCTTTGGATCCTTGAAGTATCTTCACTTCCTTGAAGCATCC 18932
              ****************************************************************************************************
Repeat2 19277:TGTCTCCTGTCACACTGCTGTGTGATACATCTCCCTATCTACAGCGGCCCACTGGTGCCCCTTTGGATCCTTGAAGTATCTTCACTTCCTTGAAGCATCC 19376

Repeat1 18933:TACTTTCAAGTCAATGCCAAGGATACACAGAGCCTCTGGACCGGTCACTTCTCCCACTTGTTTCTCATTGGGCTCCCCTCAGCTCCCACCATGGCAATTG 19032
              ****************************************************************************************************
Repeat2 19377:TACTTTCAAGTCAATGCCAAGGATACACAGAGCCTCTGGACCGGTCACTTCTCCCACTTGTTTCTCATTGGGCTCCCCTCAGCTCCCACCATGGCAATTG 19476

Repeat1 19033:GGATCCCCCCGTCACTCTGGAAATCCAGATGTGTCCTGTCCCCTTGTACCCCGATGCCATTAGGGGTCACTGTGCACCAGTGTCCACCAGGGCCTTATAC 19132
              ****************************************************************************************************
Repeat2 19477:GGATCCCCCCGTCACTCTGGAAATCCAGATGTGTCCTGTCCCCTTGTACCCCGATGCCATTAGGGGTCACTGTGCACCAGTGTCCACCAGGGCCTTATAC 19576

Repeat1 19133:TTCCATGGGTCTGATGTGCCAGGCCATCGCATCTGTTTGTCCCTTTCTTCTGCCCAGCCGGAGGCAGGACCCCTAGTCCTGATCAGAGCATTCGTTACCC 19232
              ****************************************************************************************************
Repeat2 19577:TTCCATGGGTCTGATGTGCCAGGCCATCGCATCTGTTTGTCCCTTTCTTCTGCCCAGCCGGAGGCAGGACCCCTAGTCCTGATCAGAGCATTCGTTACCC 19676

Repeat1 19233:GACTGCTGCAATTGCAAACCAGAAGCCTTTTCACCCCGAGAGGAGCCCCTGCCTCTCTCATCCAGGCTACGCTGCAAGGACTAGTCACAGGTACGATCCT 19332
              ****************************************************************************************************
Repeat2 19677:GACTGCTGCAATTGCAAACCAGAAGCCTTTTCACCCCGAGAGGAGCCCCTGCCTCTCTCATCCAGGCTACGCTGCAAGGACTAGTCACAGGTACGATCCT 19776

Repeat1 19333:ACTACAGTCTGACACGCTCTTTCCGTGGTGCTGGGCGTGAGGTTCGAGCCCACGTGGACATCTGTCCACTGGATCAGGATGAGAAGGAGGAGACGTGTCC 19432
              ****************************************************************************************************
Repeat2 19777:ACTACAGTCTGACACGCTCTTTCCGTGGTGCTGGGCGTGAGGTTCGAGCCCACGTGGACATCTGTCCACTGGATCAGGATGAGAAGGAGGAGACGTGTCC 19876

Repeat1 19433:TCAGCCCATCTACTGCGCCTGGGGAATTGCTTGACGGCAACTGGAGCAGGAATCTTCCTGCATGGTCCCTTTTTAGCCACTGTTCTTCCCCACAATTCTT 19532
              ****************************************************************************************************
Repeat2 19877:TCAGCCCATCTACTGCGCCTGGGGAATTGCTTGACGGCAACTGGAGCAGGAATCTTCCTGCATGGTCCCTTTTTAGCCACTGTTCTTCCCCACAATTCTT 19976

Repeat1 19533:GCACGTGGGCTTCCAGCAGCGAGGCGGGTGCACCAGCACGCTGCCACGTGTCCCCTGCATGGGCACACAGACAGAGCCCTACGAGGTGGCATTTGCCCAC 19632
              ****************************************************************************************************
Repeat2 19977:GCACGTGGGCTTCCAGCAGCGAGGCGGGTGCACCAGCACGCTGCCACGTGTCCCCTGCATGGGCACACAGACAGAGCCCTACGAGGTGGCATTTGCCCAC 20076

Repeat1 19633:GGTCCCCATTCCGCTGAGCAGGCTCCTGGTAGCTGAGATGCTGGCCTGTAGGCGCAAGGAGGGAGATTCTCCACCTTCTCTTCCCGTGGCCGGACCTTGG 19732
              ****************************************************************************************************
Repeat2 20077:GGTCCCCATTCCGCTGAGCAGGCTCCTGGTAGCTGAGATGCTGGCCTGTAGGCGCAAGGAGGGAGATTCTCCACCTTCTCTTCCCGTGGCCGGACCTTGG 20176

Repeat1 19733:GGAACAGTCTGGCCACAGCCGAGACAAAGACCCATGGTGGCAAGGGGGAACAGAAGTTCTCCTCGAATCACTGGCACTGGCACACCAGTGCACGGACAGC 19832
              ****************************************************************************************************
Repeat2 20177:GGAACAGTCTGGCCACAGCCGAGACAAAGACCCATGGTGGCAAGGGGGAACAGAAGTTCTCCTCGAATCACTGGCACTGGCACACCAGTGCACGGACAGC 20276

Repeat1 19833:TGGTGCCTCCATGTCTGCCCAGTCCACCACGGCCGGGTTACTGGCGCACATCGGCGTGCTCCGTACAAACATCCGCCATAGAGACTGTGCGTGCCAGATG 19932
              ************************************************************************* **************************
Repeat2 20277:TGGTGCCTCCATGTCTGCCCAGTCCACCACGGCCGGGTTACTGGCGCACATCGGCGTGCTCCGTACAAACATCTGCCATAGAGACTGTGCGTGCCAGATG 20376

Repeat1 19933:CCATCCCCATCTTTGGGGGCCCGATTATTGTCCGGGTCACTGTAGACCACCACGAATGCCCTCAGATACTGGATATCCTCCTCCCTGGTAGACCACTTAC 20032
              ****************************************************************************************************
Repeat2 20377:CCATCCCCATCTTTGGGGGCCCGATTATTGTCCGGGTCACTGTAGACCACCACGAATGCCCTCAGATACTGGATATCCTCCTCCCTGGTAGACCACTTAC 20476

Repeat1 20033:CTCGCTGGGTTACAGCATCTTCCTTGAGAGGATACTTTGCCTTCGCGCTGTCACGGAGGTACACTAAAACTAAGTGAAACGAGGGACAAGCTCAAACAAC 20132
              ****************************************************************************************************
Repeat2 20477:CTCGCTGGGTTACAGCATCTTCCTTGAGAGGATACTTTGCCTTCGCGCTGTCACGGAGGTACACTAAAACTAAGTGAAACGAGGGACAAGCTCAAACAAC 20576

Repeat1 20133:TTTATTTCACAAAACAGCTAAAACCACACGGCAGAAACCAATGAGGAACAGGGGTAAACCGAAACCAATCAACACTCCGATAATGTCACACATACCAGGT 20232
              ****************************************************************************************************
Repeat2 20577:TTTATTTCACAAAACAGCTAAAACCACACGGCAGAAACCAATGAGGAACAGGGGTAAACCGAAACCAATCAACACTCCGATAATGTCACACATACCAGGT 20676
```

```
Repeat1 20233:TCGAGGTGTCAGTCGGGGTGTTGTCACTACACAGGAACAGACACAGGAATGATGATCCAAAGTGCGCACACACACTCACAAAGGGGGAACTGGCTCTCTG 20332
              ******************************** *******************************************************************
Repeat2 20677:TCGAGGTGTCAGTCGGGGTGTTGTCACTACACGGGAACAGACACAGGAATGATGATCCAAAGTGCGCACACACACTCACAAAGGGGGAACTGGCTCTCTG 20776

Repeat1 20333:TCTCTCTTGTGGGTACCCAGAAGATAGAATCGGTCATGTGGGGGGGTGAGGACTCACTCAAGGATATATCCAGCAGAAATCACTCAGCACAAGAGGTGAG 20432
              ****************************************************************************************************
Repeat2 20777:TCTCTCTTGTGGGTACCCAGAAGATAGAATCGGTCATGTGGGGGGGTGAGGACTCACTCAAGGATATATCCAGCAGAAATCACTCAGCACAAGAGGTGAG 20876

Repeat1 20433:GGCACTCAGTCCTGGGAAGTTTCCTCAGGCGGCGTCCCAACCCGAGGGGAGAGGTCCTGAACACAGGCCCACTGCTCTGAGACCACACTCAAAGGCGGTC 20532
              ****************************************************************************************************
Repeat2 20877:GGCACTCAGTCCTGGGAAGTTTCCTCAGGCGGCGTCCCAACCCGAGGGGAGAGGTCCTGAACACAGGCCCACTGCTCTGAGACCACACTCAAAGGCGGTC 20976

Repeat1 20533:ACCGGCGGCACCCCATTTATACCCTGGGTTGGACCTGAGCTGTGGTCATAATTGGTCATGATCTAAAAGCTTCTTTGAGCCAAGGCACTTCATTGGCTGT 20632
              *************************************************************************** ************************
Repeat2 20977:ACCGGCGGCACCCCATTTATACCCTGGGTTGGACCTGAGCTGTGGTCATAATTGGTCATGATCTAAAAGCTTCTCTGAGCCAAGGCACTTCATTGGCTGT 21076

Repeat1 20633:AGACCCTGTCTGGTGACAAAGGGGTGATCGCCATGTTCAAAGCTCCACTCTATTGGGTGGTGGTGGGGGTGGTGGGGTGGGCAGGAGCGTACCTGTCACA 20732
              ****************************************************************************************************
Repeat2 21077:AGACCCTGTCTGGTGACAAAGGGGTGATCGCCATGTTCAAAGCTCCACTCTATTGGGTGGTGGTGGGGGTGGTGGGGTGGGCAGGAGCGTACCTGTCACA 21176

Repeat1 20733:CACGCTCAAGAAGAGCCACCTCCAGAGGTGGTGGGTGGCTGCCCCTCTTCCGACCCATCAATCCCACAGTCCCTAGCAATGCATCCCACTGCTGGGCTTC 20832
              ****************************************************************************************************
Repeat2 21177:CACGCTCAAGAAGAGCCACCTCCAGAGGTGGTGGGTGGCTGCCCCTCTTCCGACCCATCAATCCCACAGTCCCTAGCAATGCATCCCACTGCTGGGCTTC 21276

Repeat1 20833:CCACCTTCTAGTTCCAGCCTGTGCGCTCCACCATGTCAGTCACAAATCCTAAACACCGCACCATGCTGGGCTGCTAGGAAGGAAATCAACTCTTTCCCAG 20932
              ****************************************************************************************************
Repeat2 21277:CCACCTTCTAGTTCCAGCCTGTGCGCTCCACCATGTCAGTCACAAATCCTAAACACCGCACCATGCTGGGCTGCTAGGAAGGAAATCAACTCTTTCCCAG 21376

Repeat1 20933:CCAAAACCAGTCCAGCCCTCGACCTGAGGCTGCGAGAGCAGCCCAGGCACCAGGAGAGGAGGCACAGCCCGCCAGAGGGTGGGGACCAGCATTTATGGTG 21032
              ****************************************************************************************************
Repeat2 21377:CCAAAACCAGTCCAGCCCTCGACCTGAGGCTGCGAGAGCAGCCCAGGCACCAGGAGAGGAGGCACAGCCCGCCAGAGGGTGGGGACCAGCATTTATGGTG 21476

Repeat1 21033:TGTGCCAGGAAATGTACACCCAGAGAAGAAATCAGTTACAACCTCAAAATTAAAAAAAAAAAGGTAATAATTGCAGTGCCCAATTTTAAAAGCATCAGAA 21132
              ****************************************************************************************************
Repeat2 21477:TGTGCCAGGAAATGTACACCCAGAGAAGAAATCAGTTACAACCTCAAAATTAAAAAAAAAAAGGTAATAATTGCAGTGCCCAATTTTAAAAGCATCAGAA 21576

Repeat1 21133:AAGAGGAAGTTCACCCAGCCAGAGGAGCCACCAGCGTAGCCGGGGGTAGGCAGGCGCAGCGTGACAAGCTCTCTCTGCAGGCAGCTCCCAGCCTCCTCCA 21232
              ****************************************************************************************************
Repeat2 21577:AAGAGGAAGTTCACCCAGCCAGAGGAGCCACCAGCGTAGCCGGGGGTAGGCAGGCGCAGCGTGACAAGCTCTCTCTGCAGGCAGCTCCCAGCCTCCTCCA 21676

Repeat1 21233:GCACCCCTCTGCGTCCAGCCGGTGAGAGGGCAGGGGCCGCATCCAGCCACGTGCCGGCCCCTCGTCAAGCGGTTAGGCGGTGAGCCCTGCAGGGATGGGA 21332
              ****************************************************************************************************
Repeat2 21677:GCACCCCTCTGCGTCCAGCCGGTGAGAGGGCAGGGGCCGCATCCAGCCACGTGCCGGCCCCTCGTCAAGCGGTTAGGCGGTGAGCCCTGCAGGGATGGGA 21776

Repeat1 21333:GGAGGAGACAGCGTCACCCAGGGCCACCTCCCTGTCCCCAACTCTTGGCTCTGGGGGTCTCGGTGCCCCCTCCAGCAGCCTGGTGTTGCTCTGGCTGCGG 21432
              ******************************************************************************** *******************
Repeat2 21777:GGAGGAGACAGCGTCACCCAGGGCCACCTCCCTGTCCCCAACTCTTGGCTCTGGGGGTCTCGGTGCCCCCTCCAGCAGCCCGGTGTTGCTCTGGCTGCGG 21876

Repeat1 21433:GTCCCACTGAGAGAGAGAGGCATCAGCATCCACCATGCTTCGCCACCAGGCTCGGGGGCTCCCCAGAGCGCAGCGACTCGCACCCACAGCCCCTCCCAGA 21532
              ****************************************************************************************************
Repeat2 21877:GTCCCACTGAGAGAGAGAGGCATCAGCATCCACCATGCTTCGCCACCAGGCTCGGGGGCTCCCCAGAGCGCAGCGACTCGCACCCACAGCCCCTCCCAGA 21976

Repeat1 21533:CCCCCAGGCTCTACCTGCGTCCCACACCCCTCTGCCTCACTGGCTCTGTGCTTTACTCCATCTGTACTTCAGAAGGCGAATCCAGCAATGATGGCGATGA 21632
              ******************************************** *******************************************************
Repeat2 21977:CCCCCAGGCTCTACCTGCGTCCCACACCCCTCTGCCTCACTGGCCCTGTGCTTTACTCCATCTGTACTTCAGAAGGCGAATCCAGCAATGATGGCGATGA 22076

Repeat1 21633:CAGCCAGGGCGGCAACAGCCACCACCAGCACGATGGTGAACAGGATGGACTCGGCTCTGGGGGAGAGCAGGGCCATGAGGTGGGGAAGGGGAACCACCCC 21732
              ****************************************************************************************************
Repeat2 22077:CAGCCAGGGCGGCAACAGCCACCACCAGCACGATGGTGAACAGGATGGACTCGGCTCTGGGGGAGAGCAGGGCCATGAGGTGGGGAAGGGGAACCACCCC 22176
                                                         UBAψ  exon4
Repeat1 21733:AGCCCACTGCTCCACATGCCCAAGGCCACCGGGCTCACCCCACGCTAAGAGGACAGGCTCGAGCAGGCTGGTGTGCTCCACACGGCACCGGTACATGTCC 21832
              ******************************** *******************************************************************
Repeat2 22177:AGCCCACTGCTCCACATGCCCAAGGCCACCGGACTCACCCCACGCTAAGAGGACAGGCTCGAGCAGGCTGGTGTGCTCCACACGGCACCGGTACATGTCC 22276

Repeat1 21833:TTCTCCTCTGGGTGGGCCTCAATGGAGGCCCAGGTGTAGTGGGTGCTGTTGCTGTTGAGTGCGACGCTGCCCCACTCGGTGTCCTGGTCCCTGACCTCGC 21932
              ****************************************************************************************************
Repeat2 22277:TTCTCCTCTGGGTGGGCCTCAATGGAGGCCCAGGTGTAGTGGGTGCTGTTGCTGTTGAGTGCGACGCTGCCCCACTCGGTGTCCTGGTCCCTGACCTCGC 22376

Repeat1 21933:CGTCCTTCAGCCAGCTGACGGTGATGGGCCGCGGGTAGAAGCCGTAAGCGCGGCAGGACAAGGTCAGGATCCCAGGGGGCCTCCTTCCCCAACACTCGGA 22032
              ****************************************************************************************************
Repeat2 22377:CGTCCTTCAGCCAGCTGACGGTGATGGGCCGCGGGTAGAAGCCGTAAGCGCGGCAGGACAAGGTCAGGATCCCAGGGGGCCTCCTTCCCCAACACTCGGA 22476
                                                                                                      UBAψ  exon3
Repeat1 22033:CCGTGGGGGGCTCTGGGGAGGGGGTGGTGGTGAGGGCCGGCACACGCACCCGCGCCGGCCGGTCCCCAGGGTCCCCGTCTCACCCTCACCTTCCTCTCCA 22132
              ****************************************************************************************************
Repeat2 22477:CCGTGGGGGGCTCTGGGGAGGGGGTGGTGGTGAGGGCCGGCACACGCACCCGCGCCGGCCGGTCCCCAGGGTCCCCGTCTCACCCTCACCTTCCTCTCCA 22576
```

```
Repeat1 22133:GCACGGCCTGCCCATAGCTCACGTATTTCCTCAGCCACTCGATGCAGGTGTTCTCCAGCTAATGCTTCAGACTCTCAGCCACAGTCCCGGCCTCCACCCA 22232
              ****************************************************************************************************
Repeat2 22577:GCACGGCCTGCCCATAGCTCACGTATTTCCTCAGCCACTCGATGCAGGTGTTCTCCAGCTAATGCTTCAGACTCTCAGCCACAGTCCCGGCCTCCACCCA 22676

Repeat1 22233:CTTCCTCTCGCTGATTTGTGCTGCCACATCTGCCACAGTGAATGACATCGTGTCCATGTCGAAGGCGATGAAGTCCCTCCCGTCATAGGTGTCCTGCTGA 22332
              ****************************************************************************************************
Repeat2 22677:CTTCCTCTCGCTGATTTGTGCTGCCACATCTGCCACAGTGAATGACATCGTGTCCATGTCGAAGGCGATGAAGTCCCTCCCGTCATAGGTGTCCTGCTGA 22776

Repeat1 22333:TACCCCCTGGTGCTACCGTCCTCCAGGAGGTCACAGCCGAGCATGCACTGCACCGTGTGAGCCCTGAAACACAACAAGGGAGACGGGGCTGAGGGGCTGC 22432
              ****************************************************************************************************
Repeat2 22777:TACCCCCTGGTGCTACCGTCCTCCAGGAGGTCACAGCCGAGCATGCACTGCACCGTGTGAGCCCTGAAACACAACAAGGGAGACGGGGCTGAGGGGCTGC 22876

Repeat1 22433:TGCAGGCCTGGGACTTCAGTGGGGCACAGTCCCCACGGCAGCGCCCCCCACTCCAGCCCCGTGTGAGGTCTGGGGGGATCGGGCACCGGCTTGAGGTCTG 22532
              ****** ***** **************** ************ ************************* *******************************
Repeat2 22877:TGCAGGTCTGGGCCTTCAGTGGGGCACAGACCCCACGGCAGCACCCCCCACTCCAGCCCCGTGTGAGGCCTGGGGGGATCGGGCACCGGCTTGAGGTCTG 22976

Repeat1 22533:CCCTCCGTCCAGCACTCGAGGTGTCATGCCAAGCCATGATGCAGACCCTCCCCGCACACCCAGGGTGTGGCTGGGCTGGCACCCAGCCCCATGGCGCCCT 22632
              ******************************************************************************************* ********
Repeat2 22977:CCCTCCGTCCAGCACTCGAGGTGTCATGCCAAGCCATGATGCAGACCCTCCCCGCACACCCAGGGTGTGGCTGGGCTGGCACCCAGCCCCACGGCGCCCT 23076

Repeat1 22633:GGGACAGCGAGGGGCTGGGATGGGTCCTGAATGCTGGGGTGTGGGGGGAACAAGGAGATGGGGTGGGGTGGGGGGAGCCCCTCTGGCTCTGGGATGGGGG 22732
              ****************************************************************************************************
Repeat2 23077:GGGACAGCGAGGGGCTGGGATGGGTCCTGAATGCTGGGGTGTGGGGGGAACAAGGAGATGGGGTGGGGTGGGGGGAGCCCCTCTGGCTCTGGGATGGGGG 23176

Repeat1 22733:GCCTGAGCACCATGGGGAGGGCAAGGGACTGGGACATGTGGGTGGGAGCCCCTCTGGTTCTGGGGGTCTTGACTGCCGTGGTGATGGTGAGGGGCTGGGA 22832
              ********************* ******************************************************* **********************
Repeat2 23177:GCCTGAGCACCATGGGGAGGGTAAGGGACTGGGACATGTGGGTGGGAGCCCCTCTGGTTCTGGGGGTCTTGACTGCCATGGTGATGGTGAGGGGCTGGGA 23276

Repeat1 22833:CGGGGCAGCCAGTCCTGGGGTGGGGTCCTGGGCACTGGGGGGGTGGGGATGCAGTGGGCCGGGGT                                    22897
              *****************************************************************
Repeat2 23277:CGGGGCAGCCAGTCCTGGGGTGGGGTCCTGGGCACTGGGGGGGTGGGGATGCAGTGGGCCGGGGT                                    23341
```

Figure S2. Comparison of the nucleotide sequences of approximately 23-kb long repeats in the MHC class I region. Identical nucleotides between the two repeat sequences were marked with asterisk. Dashes indicate the gaps. The arrows indicate the tandem repeat sequences. The yellow shadows indicate exon 3 and exon 4 of UBA1ψ and UBA2ψgenes.

```
                 ┌→ exon 1                        ┌→ exon 2
UAA           1 MGSSWAPVLGLLLLGVLGGRAANVLHSLRYFDVAVSEPSPGVPKFMSVGYVDGNPFTYYDSETGREEPRADWMAASVDQQYWDRQTQISQ 90
UBA1pseudo    0 ------------------------------------------------------------------------------------------ 0
UBA2pseudo    0 ------------------------------------------------------------------------------------------ 0
                                      ┌→ exon 3
UAA          91 SNQQVNYVDLETLRGRYNQSRGAHTVQRMYGCDLLEDGSTRGYQQDAYDGRDFIAFDMDTMTFTAADAAAQITKRKWEEDGTEAERLKHY 180
UBA1pseudo    1 ----------------------.....C.L................T..............S..V..V....SE...V.A..V..S...* 68
UBA2pseudo    1 ----------------------.....C.L................T..............S..V..V....SE...V.A..V..S...* 68
                                        ┌→ exon 4
UAA         181 LKNTCIEWLRKYVSYGQAVLERKEPPAVRVSGKEAHGILTLSCRAYGFYPRPITISWLKDGEVRDQDTEWGSVVPNSNGSYYTWASIEAR 270
UBA1pseudo   69 .E........................T...L..RPP..................V.................AL...STH.........H 158
UBA2pseudo   69 .E.....................S..RSECW.RRPP..................V.................AL...STH.........H 158
                                        ┌→ exon 5
UAA         271 PEEKDMYRCRVEHASLPEPILLAWEPESNLLIIVLAVAVAILAVIAIIAGFAFWKYRSARASGPVSKKAVGGTQGLGAQEGLLTA*     356
UBA1pseudo  159 .............T..L..V....                                                                   182
UBA2pseudo  159 .............T..L..V....                                                                   182
```

Figure S3. Alignment of amino acid sequences of the MHC-I genes (UAA, UBA1ψ, and UBA2ψ). Identity with the UAA sequence is indicated with a dot. Dashes and asterisks indicate gaps and stop codons, respectively.

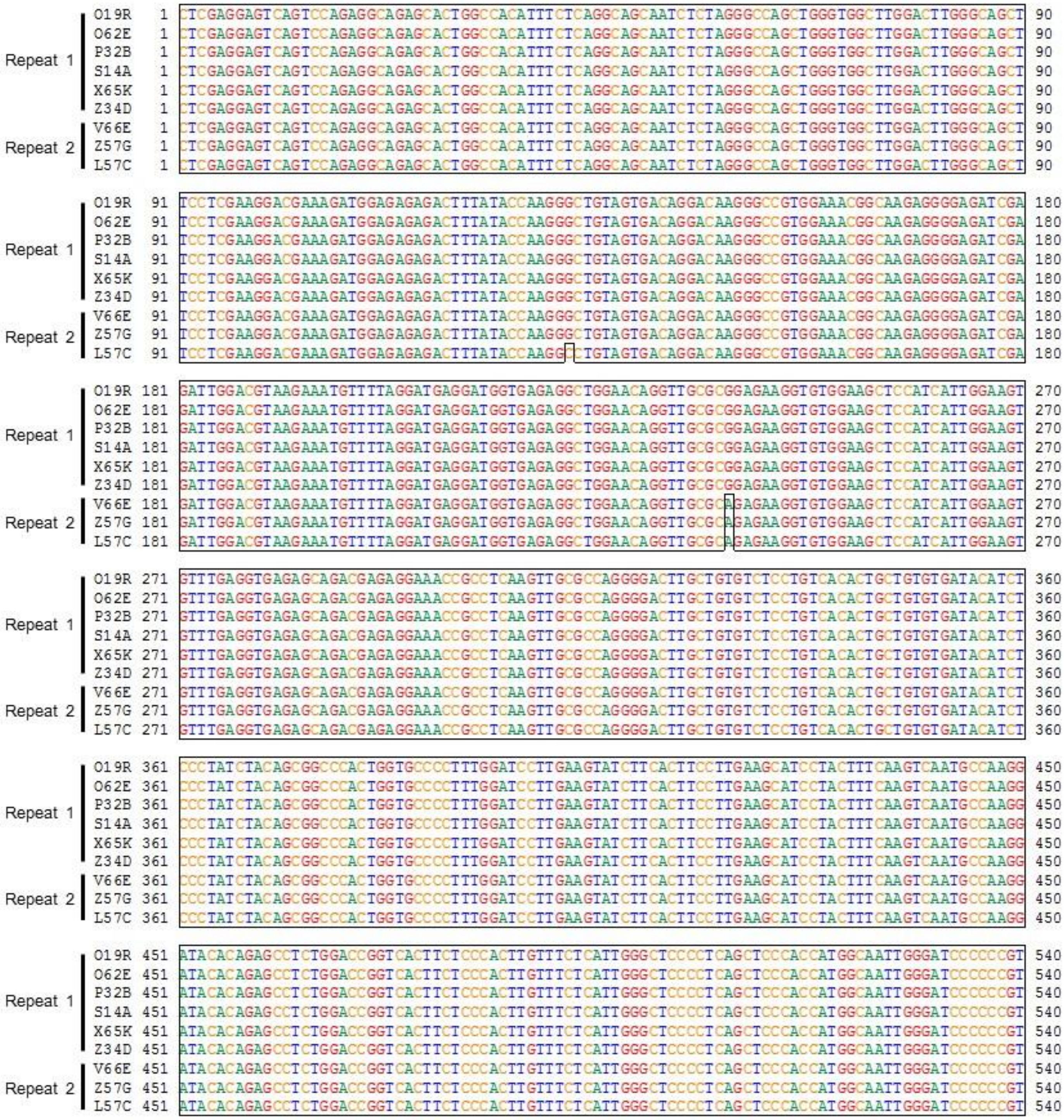

Figure S4. Alignment of 540-bp nucleotide sequences from the XhoI site among the nine phage clones. The 540-bp sequences from nine phage clones, O19R, S14A, X65K, O62E, P32B, Z34D, V66E, Z57G, and L57C were aligned. Two nucleotide substitution sites were detected. The polymorphism at nt239 suggested different loci (repeat 1: O19R, S14A, X65K, O62E, P32B, and Z34D, repeat 2: V66E, Z57G, and L57C), whereas the polymorphism at nt132 suggested two alleles within repeat 2.

```
              exon2

                 * * *        * * *          **        *        *   **   *  * **  *   *  **     **
Cibo-DAB*01 1 FFQELCNSKCEYLNGTEKVRFVQMYNYNRQQYVHFDSDVGHFVADSPLGEPDAKYWNSQPDVLEQKRGEVDRFCRHNYGVSTPFIVERR  89
Cibo-DAB*02 1 .............................E.F...................S...L.....M.DD..AA..T...Y....V...T....  89
Cibo-DAB*03 1 .....S.......................E.L.......................L.......DDR.AA..T......R..........  89
Cibo-DAB*04 1 .....S................E..IH..E.L...............................DD..AA......Y.............  89
Cibo-DAB*05 1 ..H.MIKF..............E..IH..E.F...................S...L.......DDE.AA..T...Y....V...T....  89
Cibo-DAB*06 1 ................................................................................V...T....  89
Cibo-DAB*07 1 .........................IH..E.F................................................V...T....  89
Cibo-DBB*01 1 Y..DYFKAD.YFT.......L.VR.I...E.L...............................DD..AA..T........W...T....  89
Cibo-DBB*02 1 Y..DYFKAD.YFT.........D..I...E.L...............................DDE.AA..T........W...T....  89
Cibo-DBB*03 1 Y..DYFKAD.YFT......T..D..I...E.L...............................DDE.AA..T........V...T....  89
Cibo-DBB*04 1 Y...YFKGD.YFT.......L.VR.I...E.....................S...........DDE.AA..T........W...T....  89
Cibo-DBB*05 1 Y..DYFKAD.YFT.........D..I...E.L...............................DDE.AA..T........V...T....  89
Cibo-DBB*06 1 Y...YFKGD.YFT.......L.VR.I...E.................................DD..AA..T............T....  89
Cibo-DBB*07 1 Y...YFKGD.YFT.......L.VR.I...E.....................S............R......S......R.V...T....  89
```

Figure S5. Alignment of predicted amino acid sequences of MHC-IIB exon 2 alleles from the six founders of Oriental storks in Japan. Identity with the Cibo-DAB*01 sequence is indicated with a dot. The asterisks above the sequence indicate peptide-binding residues[S1].

[S1] Brown JH. *et al*. Three-dimensional structure of the human class II histocompatibility antigen HLA-DR1. *Nature* **364**: 33-39 (1993).
